# Organic acid and sugar components accumulation and flavor associated metabolites dynamic changes in yellow- and white-fleshed seedless loquats (*Eriobotrya japonica*)

**DOI:** 10.1016/j.fochx.2023.101046

**Published:** 2023-12-06

**Authors:** Xinya Liu, Liqin Song, Baogui Xue, Zhuoheng Chi, Yuan Wang, Songqin Wen, Wenjuan Lv, Qiankun Hu, Qigao Guo, Shuming Wang, Di Wu, Guolu Liang, Danlong Jing

**Affiliations:** a Key Laboratory of Agricultural Biosafety and Green Production of Upper Yangtze River (Ministry of Education), College of Horticulture and Landscape Architecture, Southwest University, Beibei, Chongqing 400715, China; b Academy of Agricultural Sciences of Southwest University, State Cultivation Base of Crop Stress Biology for Southern Mountainous Land of Southwest University, Beibei, Chongqing 400715, China; c Jiuquan Forest Fruit Service Center, Jiuquan, Gansu 735000, China

**Keywords:** Triploid loquat, Fruit flavor, Metabolomic analysis, Carotenoids, Lipidome

## Abstract

Triploid loquats are divided into yellow- and white-fleshed cultivars. To better understand taste variations in triploid loquat fruits, we used a UPLC-ESI-QTRAP-MS/MS-based widely targeted metabolomic analysis to examine the metabolic composition of two different color fleshed triploid loquats with a sample size of 54 and external validation method within a confidence level of *P*＜0.05. We identified key flavor-related differentially accumulated metabolites using the variable importance in projection (VIP) value (VIP ≥ 1.0) and fold change ≥ 2 or ≤ 0.5. Furthermore, the results of the HPLC analysis showed that white-fleshed loquats had a low malic acid content. We also performed the UPLC-MS/MS system to investigate the carotenoids contents and lipidome in four triploid cultivars. In the fruits of white-fleshed varieties, the carotenoids contents were significantly downregulated, but the contents of most glycerolphospholipids were increased. Our results reveal the metabolomic changes between the yellow- and white-fleshed cultivars.

## Highlights

• Metabolomic analysis reveals key metabolites differences in triploid loquats.
• Key differentially accumulated metabolites involved in flavor-related variation.
• White-fleshed triploid loquats have better flavor due to lower malic acid content.
• Carotenoids contents were downregulated in the fruits of white-fleshed varieties.
• Glycerolphospholipids from the lipidome were increased in white-fleshed varieties.

Chemical compounds studied in this article.

**Table:** 

Chemical compounds	PubChem CID	Formula	Source	Class I	Class II	Information
PI(18:3_16:0)	52,927,793	C_43_H_77_O_13_P	Lipidome	Glycerol Phospholipids (GP)	Phosphatidy-linositol (PI)	The two lipids may be biomarkers between yellow- and white-fleshed triploid loquat
PE(18:3_18:3)	9,546,735	C_41_H_70_NO_8_P	Lipidome	Glycerol Phospholipids (GP)	Phosphatidy lethanolamine (PE)	
β-carotene	5,280,489	C_40_H_56_	Determination of carotenoids	Carotenoids	Carotenes	These three carotenoids contribute to the color differences
Violaxanthin-myristate-caprate	(N/A)	C_64_H_100_O_6_	Determination of carotenoids	Carotenoids	Xanthophylls	
Lutein dilaurate	122,234,427	C_64_H_101_O_4_	Determination of carotenoids	Carotenoids	Xanthophylls	
Trigonelline	5570	C_7_H_7_NO_2_	Metabolome	Alkaloids	Alkaloids	Trigonelline is only detected in WHZY
Catechin	9064	C_15_H_14_O_6_	Metabolome	Flavonoids	Flavanols	The metabolites of flavonoids in loquats with yellow and white flesh are significantly different, and these two metabolites are related to the flavor of the fruit.
Narirutin	442,431	C_27_H_32_O_14_	Metabolome	Flavonoids	Dihydroflavone	
(S)-2-(4-aminobutanamido)-3-(1-methyl-1H-imidazol-5-yl) propanoic acid	20,849,429	C_11_H_18_N_4_O_3_	Metabolome	Organic acids	Organic acids	The DAM with the largest VIP values during the FR stages.
Malic acid	525	C_4_H_6_O_5_	HPLC	Organic acids	Organic acids	White-fleshed triploid loquats were significantly lower in malic acid than yellow-fleshed triploid loquats.

## 1 Introduction

Loquat (*Eriobotrya japonica* Lindl.) is a subtropical evergreen fruit tree belonging to the Amygdaloidea subfamily Rosaceae (Jing et al., 2023). Loquat fruits are delicious and rich in nutrients, including carbohydrates, organic acids, amino acids, vitamins, and minerals (Tian et al., 2011). They are used in the food industry to prepare juice, wine, syrups, and jams (Meng et al., 2022). Furthermore, loquat fruits and other related products contain abundant antioxidants and exhibit high bioactivity (Chinese Pharmacopoeia Commission, 2020). It was often used in ancient China to treat cough and stomach problems (Li, 1578) and is used to counteract inflammation (Jian et al., 2020), cancer, melanogenesis, acne, and other health issues (Tan et al., 2017).

Most loquats are diploids (2n = 2x = 34) and their fruits usually contain four to seven large seeds that are not conducive to processing. However, compared to diploid loquats, triploid loquats (2n = 3x = 51) are available germplasms due to their outstanding properties, such as seedless fruit, large size, and high edible fruit rate. In our laboratory, abundant triploid loquat germplasms obtained from the open-pollinated seeds of Chinese diploid cultivars have been identified. Therefore, an abundant triploid loquat material has been used for quality identification. Triploid loquats have been divided into yellow-fleshed and white-fleshed categories according to fruit color observed in the peel and flesh, similar to that of diploid loquats. Whether seeded or seedless, people prefer white-fleshed loquats to yellow-fleshed loquats because of their excellent flavor (Li, 1578).

Previous studies have often focused on diploid loquats by comparing the differences in sugar fractions (Li et al., 2017), organic acids (Chen et al., 2008), and amino acids (Zhang et al., 2013) among their varieties. However, these studies have focused only on one aspect to elaborate on the differences between yellow- and white-fleshed loquats. Although fruit quality may need to be comprehensively evaluated, a more systematic understanding of these differences is now possible because of the development of metabolomic technologies. The metabolome is a more effective approach to identifying the role of differentially accumulated metabolites (DAMs) in fruits (Alseekh et al., 2021, Zhu et al., 2018). Only one study has compared the differences in metabolome between two varieties of diploid yellow- and white-fleshed loquats (Zou et al., 2020), which identified 536 metabolites. However, further metabolomic analyses of yellow- and white-fleshed triploid loquats at different developmental stages are thus required.

In this study, we selected triploid loquats with the best fruit quality and divided the growth of loquats into different periods according to their morphology (Cai et al., 2019). We set three representative periods for sample collection. The yellow-fleshed triploid cultivar, Huajinwuhe No.1 (HJWH No.1) (Dang et al., 2019a), and the white-fleshed triploid cultivar, Wuhezaoyu (WHZY) (Dang et al., 2019b), were used as plant materials for an accurate metabolome. Furthermore, we used high-performance liquid chromatography (HPLC) to investigate the differences in various sugar components and organic acids, which are crucial for fruit quality. To further verify the differences of metabolites in the flesh of triploid loquats, we selected two yellow-fleshed triploid loquat varieties (DWX-3x and HJWH No.1) and two white-fleshed triploid loquat varieties (WHZY and HYWH No.1) and determined their carotenoids and lipids contents by using UPLC-QTRAP-MS. Through these experiments, we expected to identify the differences in flavor of yellow- and white-fleshed triploid loquats and explore the key factors responsible for these differences during fruit development. Our findings might provide new directions in identifying key flavor-related metabolites of the triploid loquat fruits, which may enable the extraction of specific compounds for industrial and pharmaceutical purposes. These results provide valuable evidence for clarifying the nutritional content of triploid loquat fruits in different stages of development.

## 2 Materials and methods

### 2.1 Plant materials

We used the model plant *Solanum Lycopersicum* (Cai et al., 2019) as a template for dividing the 4 to 6 months from post-flowering to maturity of loquat fruits into five stages: green fruit (GF, day after flower 100 days), mature green fruit (MG, day after flower 120 days), color turning (CT, day after flower 140 days), orange color (OC, day after flower 160 days), and fruit ripening (FR, day after flower 180 days). Fruits of yellow- and white-fleshed triploid loquats were selected in our loquat germplasm resource nursery of Southwest University, including the two varieties of yellow-fleshed Huajinwuhe No.1 (HJWH No.1) and white-fleshed Wuhezaoyu (WHZY).

To validate the widely targeted metabolomic data, we selected two additional triploid loquat varieties (yellow-fleshed DWX-3x and white-fleshed HYWH No.1) for the determination of fruit quality, carotenoids, and lipidome. All triploid loquat trees were more than 10 years old, had approximately the same growth pattern, were free from pests and diseases, and had a consistent management level. The plant material information and sampling standard for these four varieties are shown in Supplementary Table S1. Fruit samples were collected during the three periods, including GF, CT, and FR. The peel and pulp were separated, frozen immediately in liquid nitrogen, and stored at −80 °C until used.

### 2.2 Metabolite extraction for metabolome

Each individual triploid loquat fruit flesh was freeze-dried under a vacuum (SCIENTZ-12 N/D, Xinzhi Co., Ltd., Ningbo, China) and ground into a fine powder using a mixing mill (MM400; Verder Shanghai Instruments and Equipment Co., Ltd., Shanghai, China) with zirconia beads at 30 Hz for 1.5 min. The powder was processed using a previously described method, with some modifications (Zhu et al., 2018). First, 100 mg powder from each individual fruit was extracted with 0.6 mL of 70 % aqueous methanol solution. The extract was then incubated overnight at 4 °C. Each sample, comprising powdered mixtures from three individual fruits, underwent an extraction process. Three biological replicates were used for each such sample. After centrifugation (10,000 × g, 10 min) at 4 °C, the extracts were absorbed on a CNWBOND carbon-GCB solid phase extraction column (250 mg, 3 mL, ANPEL, Shanghai, China) and filtered through a 0.22 μm nylon syringe filter (SCAA-104, ANPEL, Shanghai, China) for ultra-performance liquid chromatography-tandem mass spectroscopy (UPLC-MS/MS) analysis (Zhu et al., 2018).

### 2.3 UPLC conditions and ESI-QTRAP-MS/MS analysis

Fruit extracts were analyzed using a UPLC-ESI-MS/MS system (Shimadzu UFLC SHIMADZU CBM30A system; Shimadzu Corp., Kyoto, Japan; MS, Applied Biosystems 4500 QTRAP, SCIEX China, Beijing, China). Analytical conditions were: UPLC column, Agilent SB-C_18_ (1.8 µm, 2.1 mm × 100 mm); the mobile phase comprised solvents A (0.1 % formic acid in pure water) and B (acetonitrile). The samples were analyzed using a gradient program that employed 95 % A and 5 % B as starting conditions. Within 9 min, a linear gradient of 5 % A and 95 % B was programmed, and the composition of 5 % A and 95 % B was maintained for 1 min. Subsequently, 95 % A and 5.0 % B were adjusted in 1.10 min and kept for 2.9 min.

Linear ion trap (LIT) and triple quadrupole (QQQ) scans were conducted using a triple quadrupole-linear ion trap mass spectrometer (QTRAP), API 4500 QTRAP UPLC/MS/MS System, with an ESI Turbo Ion-Spray interface. This system operated in both positive and negative ion modes and was controlled through Analyst 1.6.3 software (AB Sciex). The ESI source operation parameters included a source temperature of 550 °C and ion spray voltage (IS) of 5500 V for positive ion mode and −4500 V for negative ion mode. The ion source gases I (GSI), II (GSII), and curtain gas (CUR) were regulated at 50, 60, and 30.0 psi, respectively, while the collision gas (CAD) was set to a high level. Mass calibration and instrument tuning were achieved using 10 and 100 μmol/L polypropylene glycol solutions in QQQ and LIT modes, respectively. QQQ scans were captured as multiple reaction monitoring (MRM) experiments with the collision gas (nitrogen) maintained at 5 psi. Individual MRM transitions underwent delustering potential (DP) and collision energy (CE) optimization. The MRM transitions monitored were specific to each time and matched to the metabolites eluted during that period.

Following the acquisition of these ion signals, based on the self-built database MWDB (Metware Database, https://www.metware.cn/, which was provided by Wuhan Metware Biotechnology Co., Ltd., China), the substance was characterized according to the secondary spectrum information. During the analysis, isotope signals, repeated signals, and other more recent signals were removed. Repetitive signals from fragment ions of large molecular weight species. The metabolomic detection procedure adhered to the European Union standards (2002/657/EC), with each precursor ion and daughter ion being assigned 1 and 1.5 points, respectively. Consequently, a minimum of two pairs of ions is required to accurately identify the target substance in LC-MS/MS. After obtaining the metabolite spectrum analysis data from different samples, all material spectrum peaks are integrated by peak area, and the spectrum peaks of the same metabolite in different samples are corrected by integration (Alseekh et al., 2021, Zhu et al., 2018).

### 2.4 Detection of sugars and organic acids by HPLC

The sugars and organic acids were extracted using the method described by Toker et al. (2013) with some improvements. To extract organic acids the pulp was mixed with ultra-pure water (v/v = 20/100), and homogenized in an ice bath for 1 min. We used the ultrasonic cleaning instrument (KQ5200E, Kunshan Shumei Instrument, Kunshan, China) to sonicate the mixed samples with 250 kW and centrifuged at 3000 × g for 10 min at 4 °C (Sorvall Legend Micro 17 Centrifuge, Thermo Fisher Scientific, Waltham, MA, USA). The supernatant was then filtered through a 0.45-µm membrane filter (Jinteng Co., Ltd., Tianjin, China). The organic acids in the supernatant were analyzed using an LC-20AT model HPLC system (Shimadzu Co., Kyoto, Japan) with a diode array detector (DAD, SPD-M20A, Kyoto, Japan). Organic acids were separated using an Inertial C_18_ column (5 μm, 250 mm × 4.6 mm, GL Science, Japan). The elution was at 30 °C with an isocratic flow of 2 % KH_2_PO_4_, pH 2.52 adjusted with phosphoric acid, as a mobile phase with a flow rate of 0.8 mL·min^−1^ with injection volumes of 10 µL. The chromatogram was continuously monitored at 210 nm throughout the elution. The contents of organic acids were calculated from the peak area via analytical interpolation of the calibration curve using a standard external method and were expressed in mg/100 g of fresh weight (FW).

The sugar content of the fruits was analyzed using an LC 20 AT model HPLC system (Shimadzu Co., Kyoto, Japan) with a refractive index detector (RID-20A). The sugars were separated using an inertial NH_2_ column (5 μm, 250 mm × 4.6 mm; GL Science, Japan). Elution was performed at 25 °C with an isocratic flow of acetonitrile: water (v/v = 70/30) at a flow rate of 1.0 mL·min^−1^ with 10 µL injection volumes. Sugar content was determined based on the standard external method from the peak area by analytical interpolation of a standard calibration curve and expressed as a percentage. Six biological replicates were included for the determination of different soluble sugars and organic acids.

### 2.5 The determination of fruit quality and color parameter

Fruit flesh was squeezed into juice to determine total soluble solids (TSS) and titratable acidity (TA) using a handheld digital refractometer (PR-101R, Atago, Japan). The exterior color of the flesh and peel of loquat fruits was determined using a color reader (CR-10 Plus; Konica Minolta, Inc., Japan). Parameters L*, a*, b*, c, h, and color contribution index (CCI) were determined by measuring the three surfaces of each loquat fruit and calculating the average size (Lindner, 1944). Three independent biological replicates were used.

### 2.6 The determination of carotenoids

To investigate the causes of color differences between yellow- and white-fleshed triploid loquats, we have determined the carotenoids in the pulp samples. The samples were freeze-dried, ground into powder (30 Hz, 1.5 min), and stored at −80 °C until used. Fifty mg powder was added into 0.5 mL mixed solution of *n*-hexane: acetone: ethanol (1:1:1, v/v/v), and vortexed for 20 min. The supernatants were collected after centrifuged at 20000 × g for 5 min at 4 °C. The residue was re-extracted by repeating the above steps again under the same conditions. These supernatants were then evaporated to dryness and reconstituted in 100 μL dichloromethane. The solution was filtered through a 0.22 μm membrane filter for LC-MS/MS analysis.

The sample extracts were analyzed using a UPLC-APCI-MS/MS system (UPLC, ExionLC™ AD, https://sciex.com.cn/; MS, Applied Biosystems 6500 Triple Quadrupole, https://sciex.com.cn/). LIT and QQQ scans were acquired on a QTRAP® 6500 + LC-MS/MS System, equipped with an atmospheric pressure chemical ionization (APCI) Heated Nebulizer, operating in positive ion mode, and controlled by Analyst 1.6.3 software (Sciex). The analytical conditions and carotenoid contents were detected by Metware (https://www.metware.cn/) based on the AB Sciex QTRAP 6500 LC-MS/MS platform (Hadjipieri et al., 2017). The absolute carotenoid contents were determined by substituting each detected peak area into their respective linear standard curve equations (Supplementary Table S6).

### 2.7 The determination of lipids

To further expand metabolomic data, we carried out the determination of lipidome using a UPLC-ESI-MS/MS system (Sun et al., 2022). Firstly, we extracted the lipids from loquat fruit flesh using a solvent system of methanol, methyl *tert*-butylether (MTBE). In more detail, the sample was thawed, 20 mg dry sample was added to the 2 mL centrifugal tube and put in 1 steel bead (internal diameter about 4 mm). The 1 mL lipid extract (MTBE: MeOH = 3:1) was added and vortexed for 30 min. Then, 300 μL ultra-pure water was added and vortexed for 1 min, Leaving the sample at 4 °C for 10 min. After centrifuging for 3 min at 16000 × g at 4 °C, 400 μL supernatant was transferred to a 1.5 mL centrifuge tube and concentrated until completely dry at 20 °C. 200 μL lipid complex solution (ACN: IPA = 1:1) was added to redissolve, after vortexed for 3 min, and centrifuged for 3 min at 16000 × g at 4 °C. Finally, 120 μL reconstituted solution was collected for LC-MS/MS analysis. Three independent biological replicates were used.

The extracts were analyzed using an LC-ESI-MS/MS system (UPLC, ExionLC AD, https://sciex.com.cn/; MS, QTRAP® 6500 + System, https://sciex.com/). The parameters of analytical conditions were as follows: UPLC column (2.6 μm, 2.1 mm × 100 mm i.d., Thermo Accucore™C30); solvent system, A: acetonitrile/water (60/40 v/v, 0.1 % formic acid, 10 mmol/L ammonium formate), B: acetonitrile/isopropanol (10/90 v/v, 0.1 % formic acid, 10 mmol/L ammonium formate); gradient program, A/B (80:20 v/v) at 0 min, 70:30 v/v at 2.0 min, 40:60 v/v at 4 min, 15:85 v/v at 9 min, 10:90 v/v at 14 min, 5:95 v/v at 15.5 min, 5:95 v/v at 17.3 min, 80:20 v/v at 17.3 min, 80:20 v/v at 20 min; flow rate, 0.35 mL/min; temperature, 45 °C; injection volume: 2 μL. The effluent was alternatively connected to an ESI-triple quadrupole-linear ion trap (QTRAP)-MS (Sun et al., 2022).

LIT and QQQ scans were acquired on a QTRAP® 6500 + LC-MS/MS System, equipped with an electrospray ionization (ESI) Turbo Ion-Spray interface and controlled by Analyst 1.6.3 software (Sciex). The ESI source operation parameters were as follows: ion source, turbo spray; source temperature 500 °C; ion spray voltage (IS) 5500 V (Positive), −4500 V (Negative); Ion source gas 1 (GS1), gas 2 (GS2), curtain gas (CUR) was set at 45, 55, and 35 psi, respectively. Instrument tuning and mass calibration were performed with 10 and 100 μmol/L polypropylene glycol solutions in QQQ and LIT modes, respectively. QQQ scans were acquired as MRM experiments with collision gas (nitrogen) set to 5 psi. DP and CE for individual MRM transitions were done with further DP and CE optimization. A specific set of MRM transitions was monitored for each period according to the metabolites eluted within this period. The lipid contents of the plant were detected by Metware (https://www.metware.cn/) based on the AB Sciex QTRAP 6500 LC-MS/MS platform.

### 2.8 Chemicals and reagents

HPLC-grade acetonitrile methanol (MeOH), acetone (AC), *N*-hexane, acetonitrile (ACN), acetic acid (AcOH), isopropanol (IPA), ethanol (EtOH), chloroform, dichloromethane (CH_2_C_l2_), and methyl *tert*-butyl ether (MTBE) were purchased from Merck (Darmstadt, Germany). HPLC-grade formic acid (FA) and ammonium formate (AmFA) were purchased from Sigma-Aldrich (St. Louis, MO. USA). AR-grade NaCl, KOH, and HPLC-grade butylated hydroxytoluene (BHT), KH_2_PO_4,_ and phosphoric acid were purchased from Aladdin (Shanghai Aladdin Biochemical Technology Co., Ltd., China). All the lipids, sugars, organic acids, carotenoids standards, and other standards used in widely targeted metabolome were purchased from Sigma-Aldrich (St Louis, MO, USA) and BOC (NY, USA). Ultra-pure water was obtained by a Milli-Q system (Millipore, Billerica, MA, USA) and used in all experiments.

### 2.9 Statistical data analysis and graphic production

Data within different groups of single fruit cultivar groups were analyzed using analysis of variance (ANOVA), and the significant differences were compared using Duncan’s multiple range test. Data within different groups of different colors were analyzed by Independent Samples *t*-test. Hierarchical cluster analysis (HCA), principal component analysis (PCA), and orthogonal partial least squares discriminant analysis (OPLS-DA) were performed using SIMCA v.14.1 (Peng et al., 2021). The sweetness index is calculated by the formula: Sweetness index = 1.00 × Glucose + 2.30 × Fructose + 1.35 × Sucrose (Keutgen and Pawelzik, 2007).

The data of metabolome, HPLC, carotenoids contents, and lipidome were transformed by the min–max method for statistical analysis to improve normality and normalized in creating graphics such as heatmaps (Han et al., 2012). The heatmaps and Venn diagram in this study were generated using the TBtools (Chen et al., 2020).

## 3 Results

### 3.1 Morphological characterization of the fruits of triploid loquat

**Fig. 1:**
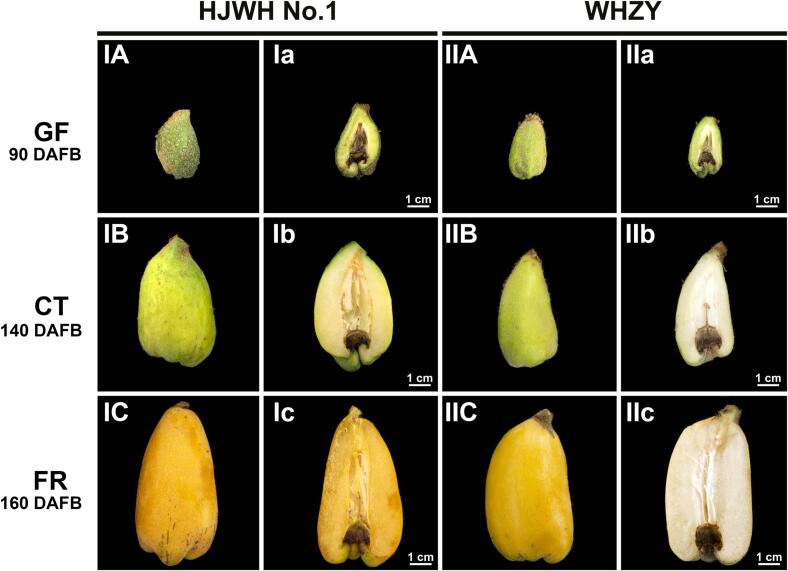
Morphological changes in yellow- and white-fleshed triploid loquat fruits across three developmental stages: green fruit (GF), color turning (CT), and fruit ripening (FR). The morphology of HJWH No.1 is illustrated at GF (IA and Ia), CT (IB and Ib), and FR (IC and Ic) stages. Similarly, the morphology of WHZY is depicted at GF (IIA and IIa), CT (IIB and IIb), and FR (IIC and IIc) stages. The transition from the GF stage to the CT stage took approximately 50 days, owing to the slower growth rate of the fruit during the colder winter months of January and February. Scale bars represent 1 cm. (For interpretation of the references to color in this figure legend, the reader is referred to the web version of this article.)

Peel and flesh samples were collected from the GF, CT, and FR stages of the two varieties in the triploid loquats. For efficient comparative analysis, we captured their photographs and measured the color parameters (Fig. 1) (Lindner, 1944). As the loquat fruit developed, the L* and b* values of the peel did not significantly differ considerably between different periods. The a* value increased and varied significantly among the different stages. In color comparison between yellow-fleshed (HJWH No.1) and white-fleshed (WHZY) loquats, L*, a*, and b* values were significant differences in the FR stage only. HJWH No.1 had relatively high a* values for peel and flesh, while the b* value of the pulp of WHZY was significantly high (Supplementary Fig. S1).

### 3.2 Identification of total metabolites in triploid loquats

**Fig. 2:**
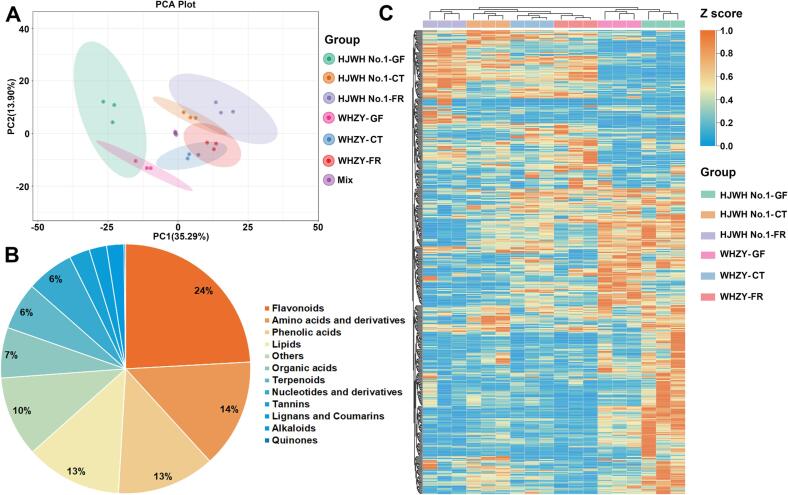
(A) PCA analysis of metabolites extracted from yellow- and white-fleshed triploid loquat fruits. An equal mixture of fruit samples was used for quality control. (B) Categorization of the 489 metabolites detected in the chestnut samples. (C) Cluster analysis of the metabolites extracted from yellow- and white-fleshed triploid loquat fruits at the GF, CT and fruit ripening FR stages. To enhance clarity of data presentation, the heatmap displays the *Z-*score from the processed data. Downregulated and upregulated differentially accumulated metabolites are represented in blue and yellow, respectively. (For interpretation of the references to color in this figure legend, the reader is referred to the web version of this article.)

By determining the metabolite profiles of yellow- and white-fleshed triploid loquats, the total metabolites were extracted in fruits at the GF, CT, and FR stages and their profiles were investigated. The sample size for the metabolome research consists of 54 individual fruit flesh samples. The population comprises six accessions that encompass two triploid loquat cultivars, across three stages of fruit development and are represented by three biological and three technical replicates. The overlapping display analysis of the total ion current (TIC) results for different quality control (QC) samples is shown in Supplementary Fig. 2A, B. There is a high degree of overlap in the TIC curves for different QC samples, indicating that the evaluation results are repeatable and reliable. Six samples were grouped and located in the center of the PCA plot, indicating high reliability and repeatability of the results (Fig. 2A). The first principal component (PC1) efficiently separated different groups at different stages of fruit development, with a 35.29 % variance contribution, and the groups with different fruit colors were mainly separated by the second principal component (PC2), with a value of 13.9 %. The PCA results of the four groups of samples showed that loquats with different treatments were separated, which indicated that the metabolic differences were significant, corresponding to the observation of the color of the flesh and other fruit developmental stages.

**Fig. 3:**
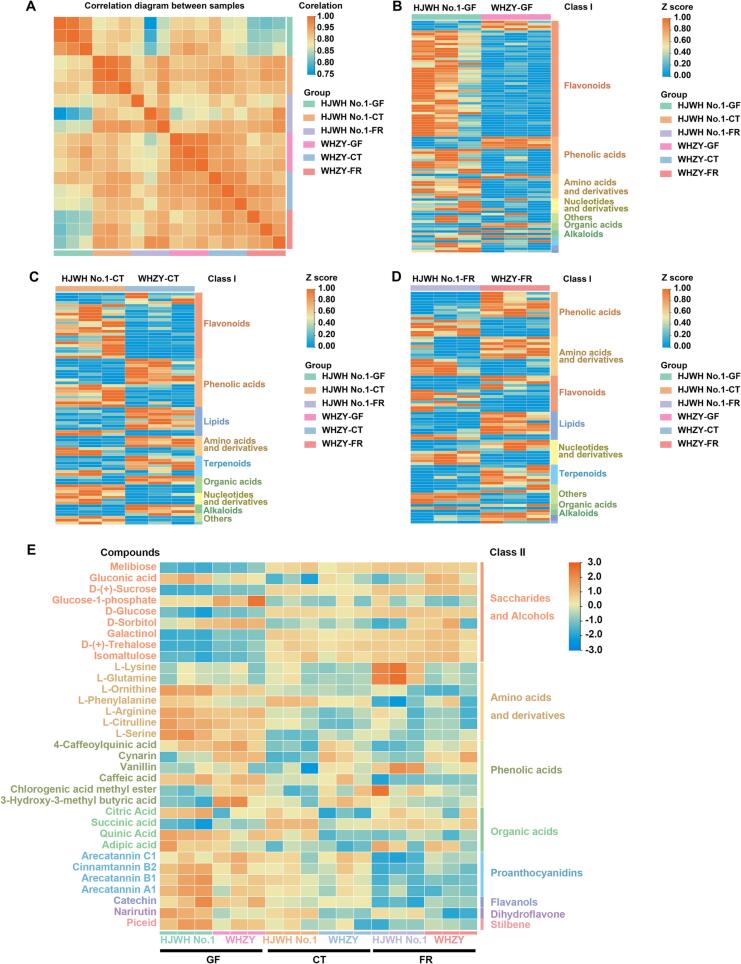
(A) Heatmap depicting the correlation among various samples, focusing on the metabolites present in the yellow-fleshed HJWH No.1 and white-fleshed WHZY loquat cultivars. (B), (C), and (D) represent the GF, CT, and FR stages, respectively. (E) Heatmap illustrating the distribution of flavor-related metabolites in yellow- and white-fleshed loquats across different developmental stages. (For interpretation of the references to color in this figure legend, the reader is referred to the web version of this article.)

A total of 489 metabolites were identified and divided into 12 classes: 118 flavonoids, 69 amino acids and their derivatives, 62 phenolic acids, 32 organic acids, 30 nucleotides, and products, 28 free fatty acids, 27 carbohydrates, and other metabolites (Fig. 2B) The information of the metabolites has been summarized in Supplementary Table S2 following the format by Alseekh et al.(2021). The accumulation pattern of metabolites among loquat samples was visualized using HCA analysis (Fig. 2C) and showed that some metabolites had an obvious tendency to shift during fruit development. We further plotted the scores using OPLS-DA to analyze pairs of two factors: different fruit colors and fruit development stages. The OPLS-DA score plot showed a significant separation among the different comparison groups (Supplementary Fig. S3). The correlation heatmap between samples of WHZY and HJWH No.1 from different stages showed that their intergroup differences were greater than the intragroup differences (Fig. 3A).

### 3.3 Identification of DAMs in triploid loquat

To effectively identify the differences in metabolites between yellow- and white-fleshed triploid loquats, we analyzed DAMs at each developmental stage. First, the values of metabolites with a fold change ≥ 2 (upregulated) or ≤ 0.5 (downregulated) were selected. These metabolites were then screened using the variable importance in projection (VIP) value (VIP ≥ 1.0) from the OPLS-DA model and these data were tested for *P*-value ≤ 0.05. Eventually, 94, 81, and 83 DAMs were identified in the two loquat cultivars at the GF, CT, and FR stages, respectively. (Supplementary Table S3). Among them, 18 were upregulated, and 76 were downregulated at the GF stage (Supplementary Fig. S4A); 37 were upregulated, 44 were downregulated at the CT stage (Supplementary Fig. S4B); 50 were upregulated, and 33 were downregulated at the FR stage (Supplementary Fig. S4C). Interestingly, the number of upregulated metabolites progressively increased, whereas the number of downregulated metabolites decreased as the fruit developed.

When fruit development reached the GF stage, the number of downregulated metabolites was higher than the upregulated metabolites. These DAMs were classified into flavonoids, phenolic acids, amino acids, and their derivatives (Fig. 3B). At the CT stage, the downregulated metabolites remained concentrated in the flavonoid class. However, many of the upregulated metabolites appeared to focus on the classes of phenolic acids, lipids, and amino acids and their derivatives (Fig. 3C). Finally, most of the DAMs of lipids and terpenoids were upregulated during the FR stage, and the DAMs in these lipid classes are primarily belonging to the glycerol phospholipids class (Supplementary Table S3). WHZY had relatively more upregulated metabolites distributed in different classes (Fig. 3D). With fruit development, the proportion of upregulated DAMs gradually increased from 19.15 % to 60.24 % in the metabolite comparison between HJWH No.1 and WHZY. We then matched these DAMs in different groups with the Kyoto Encyclopedia of Genes and Genomes (KEGG) database to obtain information regarding the pathways involving these metabolites. DAMs by comparing HJWH No.1 and WHZY at the GF stage were mainly annotated and enriched in the flavone and flavanol biosynthesis and purine metabolism pathways (Supplementary Fig. S4D). During the CT stage, DAMs were mainly annotated and enriched in plant hormone signal transduction, C5-branched dibasic acid metabolism, and isoquinoline alkaloid biosynthesis pathways (Supplementary Fig. S4E). In the FR stage, the DAMs were mainly enriched in the biosynthesis of secondary metabolites, the biosynthesis of amino acids, and the ABC transporter pathways (Supplementary Fig. S4F).

### 3.4 Analysis of key DAMs related to flavor

We summarized the different metabolites associated with fruit flavor from previous studies to determine differences in flavor-related metabolites between yellow- and white-fleshed triploid loquats. We analyzed the peak areas using metabolomic data (Supplementary Table S4). In the class of saccharides and alcohols, only melibiose (PubChem CID: 400658) and gluconic acid (PubChem CID: 92283) showed significant differences, and the melibiose was more abundant in HJWH No. 1 whereas the gluconic acid was more abundant in WHZY. The l-lysine (PubChem CID: 5962) and l-glutamine (PubChem CID: 5961), belonging to the classes of amino acids and their derivatives, were significantly higher in HJWH No.1 than in WHZY. In contrast, the remainder of this class was insignificant. All four acids were present in significantly higher levels while comparing flavor metabolites of phenolic acids, 4-caffeoylquinic acid (PubChem CID: 9798666), cynarin (PubChem CID: 5281769), vanillin (PubChem CID: 1183), and villoy caffeoyl tartaric acid (PubChem CID: 6440397) in WHZY than in HJWH No.1. However, no significant differences were found among the five organic acids associated with the flavor of the metabolome. Arecatannin C1 (PubChem CID: 9876038) and cinnamtannin B2 (PubChem CID: 16130973), two tannin compounds, were significantly more abundant in WHZY than in HJWH No.1. In flavonoid compounds, catechin (PubChem CID: 9064) were significantly higher in WHZY than in HJWH No.1, and the narirutin (PubChem CID: 442431) were significantly lower. In the dynamic expression of these flavor-related metabolites, the sugar and alcohol content gradually increased with fruit development, whereas the rest of the amino acids and their derivatives, phenolic acids, organic acids, pro-anthocyanidins, flavonols, dihydro flavone, and stilbene gradually decreased with fruit development (Fig. 3E).

### 3.5 Screening for key DAMs between yellow- and white-fleshed triploid loquat

**Fig. 4:**
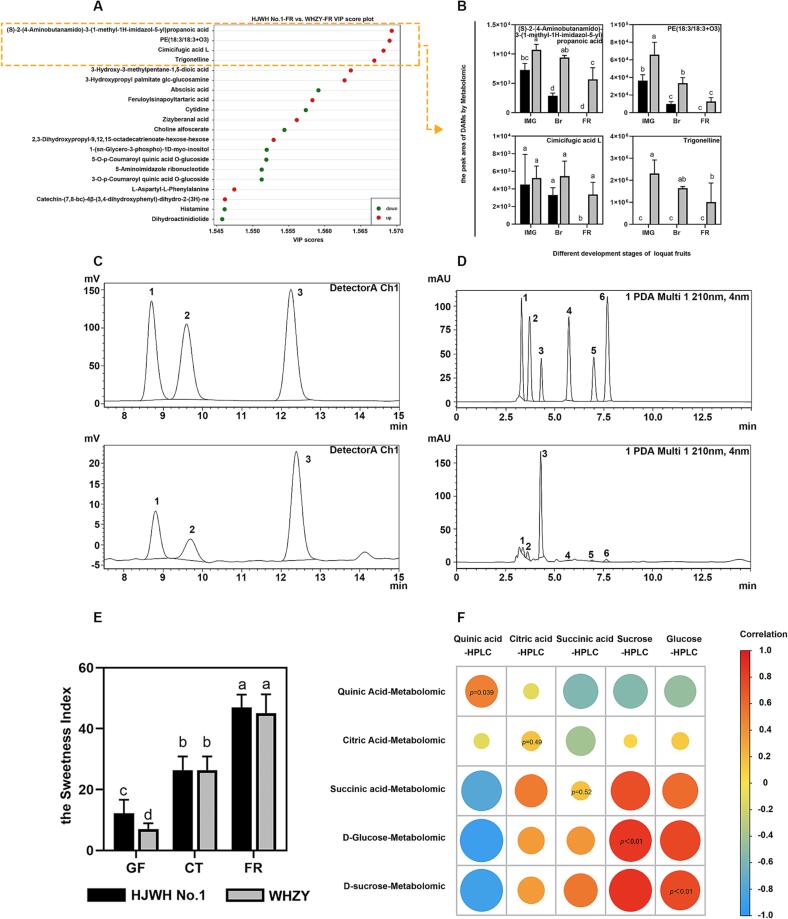
(A) VIP score diagram illustrating DAMs in the HJWH No.1-FR *vs*. WHZY-FR group comparison. (B) Column chart depicting DAMs in the HJWH No.1 *vs.* WHZY group comparison at the FR stages. Each data point represents the mean of three measured values, with bars indicating standard deviation (SD) (n = 3). Statistically significant differences are denoted by differing letters, determined using one-way ANOVA followed by Duncan’s multiple range test, with a *P* value < 0.05 marked by *. (C-D) HPLC chromatograms generated from Shimadzu’s LC-20A. (C) Peaks 1, 2, and 3 represent fructose, glucose, and sucrose, respectively. (D) Peaks 1–6 signify oxalic acid, quinic acid, malic acid, citric acid, succinic acid, and fumaric acid, respectively. (E) Sweetness index representation of HJWH No.1 and WHZY at various developmental stages. (F) Correlation heatmap of DAMs identified by metabolomic analysis and HPLC. Color depth and circle size are indicative of the correlation magnitude, with data on the circles representing the *P* value of the student’s *t*-test.

We analyzed DAMs of the two varieties at the FR stage to compare flavor differences between yellow- and white-fleshed triploid loquats. According to the highest VIP values and the OPLS-DA model (Fig. 4A, B), we selected four metabolites, including (S)-2-(4-aminobutanamido)-3-(1-methyl-1H-imidazol-5-yl) propanoic acid (PubChem CID: 20849429), PE(18:3/18:3 + O3), cimicifugic acid L (PubChem CID: 46210732), and trigonelline (PubChem CID: 5570). Analysis of the peak areas of these four metabolites in the two varieties at different stages showed that, except for trigonelline, all other metabolites showed a decreasing trend with fruit development. Their peak areas in HJWH No. 1 decreased approximately to zero at the FR stage, which differed significantly from those in WHZY. In addition, trigonelline was a unique metabolite in WHZY.

To better visualize key DAMs, we performed a Venn diagram analysis of DAMs in three comparisons of the GF, CT, and FR stages between HJWH No.1 and WHZY, respectively. We identified 14 key metabolites in the three comparison groups (Supplementary Fig. S5, Supplementary Table S5). These DAMs contained six phenolic acids, four flavonoids, one other class, one amino acid and its derivatives, and one alkaloid. Furthermore, it is also interesting to note that trigonelline is among these 14 DAMs.

### 3.6 The content of different sugar components and organic acids detected by HPLC

**Fig. 5:**
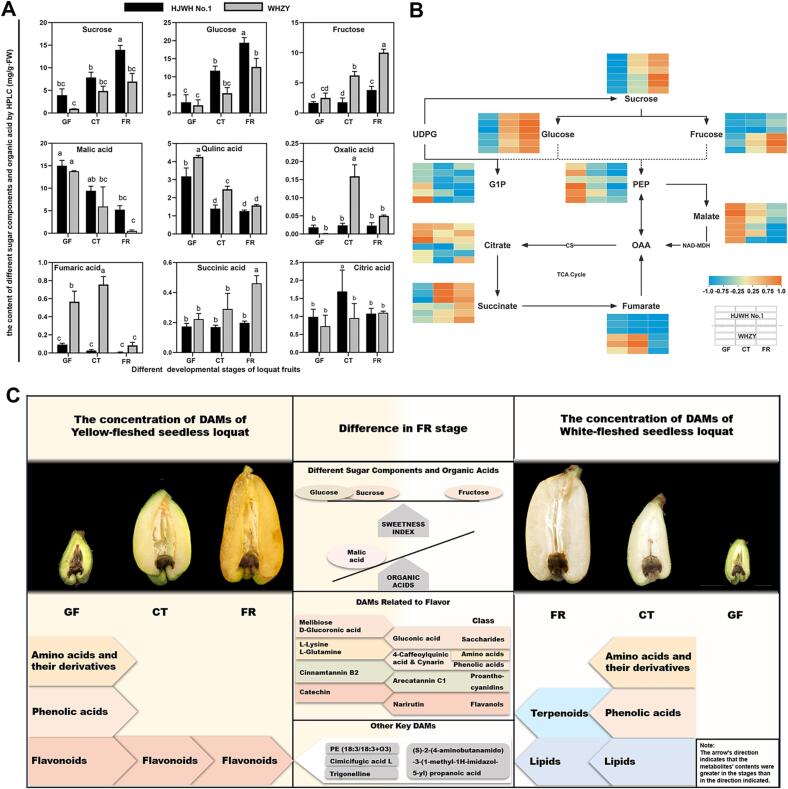
(A) Depicts bar graphs representing the presence of three sugar components and six organic acids detected by HPLC at the GF, CT, and FR stages. The analysis was conducted using six replicates and the results are presented as mean ± standard deviation (SD). Different alphabetical indicators above the bars denote statistically significant differences as determined by one-way Analysis of Variance (ANOVA), followed by Duncan’s multiple range test (*P* < 0.05). (B) Illustrates the metabolic pathways of sugar components and organic acids, integrating the results of HPLC and metabolomic analyses. The data in the heatmaps were normalized using the min–max method (Han et al., 2012). Explicatory notes on the metabolism and transport of sugar and organic acids, including glucose-1-phosphate (G1P), citrate synthase (CS), phosphoenolpyruvate (PEP), malate dehydrogenase (NAD-MDH), and oxalacetic acid (OAA) are provided. (C) Provides a schematic of the differential changes in metabolites of yellow- and white-fleshed triploid loquat during fruit development. (For interpretation of the references to color in this figure legend, the reader is referred to the web version of this article.)

To further investigate the differences in taste between yellow- and white-fleshed triploid loquats, we examined three sugar components and six organic acids from these samples using HPLC with a sample size of 36. The sample size for the metabolome study consists of 36 individual fruit flesh samples. These samples contained three stages of fruit development in two triploid loquat cultivars. The chromatograms of the standards and samples of different sugar components and organic acids are shown in Fig. 4C, D. Each peak was separated, indicating that the results were conclusive. As shown in Fig. 5A, the contents of fructose, sucrose, and glucose showed an upregulated trend, and the contents of glucose and sucrose in HJWH No.1 were significantly higher than those in WHZY. In contrast, the fructose content in WHZY was significantly higher than that in HJWH No.1. However, the sweetness index revealed no significant difference between their sweetness of the flesh at the FR stage (Fig. 4E).

The most abundant organic acids detected in triploid loquat fruits were malic acid and quinic acid, which exhibited decreasing trends during fruit development. The contents of the remaining four acids were especially low and not significantly different between developmental stages or between the yellow- and white-fleshed varieties (Fig. 5A). Malic acid was dominant in loquat fruits. In contrast, the malic acid content of the mature flesh of WHZY was significantly lower than HJWH No.1, which is the main factor affecting the sour taste. Briefly, HJWH No.1 and WHZY did not present significant differences in sweetness index; however, HJWH No.1 was relatively sourer. We then carried out a correlation analysis between these metabolites and the metabolome. The correlation analysis showed that sucrose, glucose, and quinic acid were significantly associated, whereas citric acid and succinic acid were not significantly correlated. The contents of sucrose, glucose, and quinic acid in the fruits were significantly higher than those of citric acid and succinic acid, and their data showed a strong correlation indicating reliable HPLC and metabolomic results (Fig. 4F). Subsequently, we combined the HPLC and metabolomic data and mapped them onto the metabolic maps of the sugar and acid pathways after processing with the min–max normalization method to observe their dynamic changes (Fig. 5B). The contents of sucrose, fructose, and glucose gradually increased with fruit development in both yellow- and white-fleshed loquats. The opposite trend was observed in the contents of phosphoenolpyruvate (PEP) and malic acid. In the tricarboxylic acid cycle, the levels of citric acid and succinic acid were higher in yellow-fleshed loquats than in white-fleshed loquats during all three developmental periods. However, an opposite trend was observed for the fumaric acid content. In conclusion, based on the data of widely targeted metabolome and HPLC, we synthesized a schematic diagram to encapsulate the changes and variations in metabolites between yellow- and white-fleshed triploid loquat during fruit development. (Fig. 5C).

**Fig. 6:**
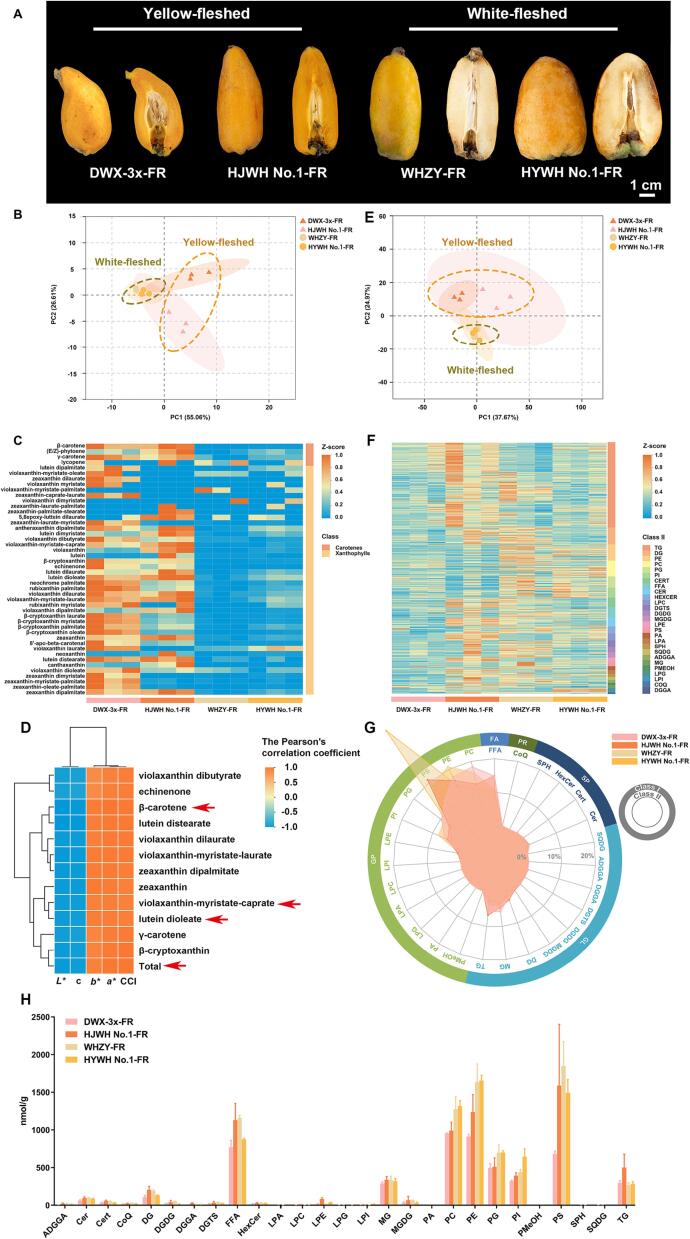
(A) Morphological depiction of two yellow-fleshed seedless triploid loquat cultivars (DWX-3x and HJWH No.1) and two white-fleshed triploid cultivars (WHZY and HYWH No.1) at the FR stage. (B, E) PCA analysis of carotenoid (B) and lipid (E) composition among the four cultivars at the FR stage. (C, F) Heatmaps of carotenoid (C) and lipid (F) content in the four loquat cultivars at the FR stage with normalization conducted using the min-max method. (D) Provides a heatmap demonstrating Pearson’s correlation coefficients between carotenoid content and color parameters (L*, a*, b*, c, h, and CCI), with the color contribution index (CCI) calculated as 1000 × a*/(L*×b*). Selection for data included in the hierarchical cluster analysis (HCA) was based on a *P*-value < 0.05 and an absolute correlation value > 0.95. The data are found in Supplementary Table S9-S10. (G) Displays a radar chart outlining the proportion of subclass lipid content within total lipid content. Full designations for Lipid class I and class II are provided in Supplementary Table S11.

To validate our findings, we analyzed fruit quality in two additional triploid loquat cultivars, yellow-fleshed DWX-3x and white-fleshed HYWH No.1, at their FR stage (Fig. 6A). Significant differences were observed in single fruit weight and firmness, while no significant differences were found in total soluble solids (TSS) and titratable acid (TA) (Supplementary Fig. S6). Various sugar components and organic acids were examined using HPLC analysis and showed significant differences in fructose, malic acid, and succinic acid. However, the sweetness indexes of the four triploid loquat varieties showed no significant difference.

### 3.7 The content differences of carotenoids between the four cultivars

Compared with white-fleshed triploid loquats, the fruits of yellow-fleshed triploid loquats exhibited significant pigmentation differences, which might be due to carotenoids contents (Cai et al., 2019). Hence, we selected four triploid loquat cultivars for the determination of carotenoid contents at the FR stage using a UPLC-QTRAP-APCI-MS/MS system. The four cultivars comprise two yellow-fleshed cultivars (DWX-3x and HJWH No.1) and two white-fleshed cultivars (WHZY and HYWH No.1). The PCA analysis (Fig. 6B) showed were classified into two distinct groups between the yellow- and white-fleshed groups. In total, 46 carotenoids were identified, of which 6 account for ∼75 % of the total carotenoid contents in the four cultivars (Fig. 6C, Supplementary Fig. S7, Supplementary Table S7-8).

Compared with white-fleshed loquats, the six carotenoids, including β-cryptoxanthin laurate (PubChem CID: 101997813), β-carotene (PubChem CID: 5280489), violaxanthin-myristate-caprate, β-cryptoxanthin oleate, β-cryptoxanthin myristate (PubChem SID: 86291094), and lutein dilaurate (PubChem CID: 122234427), were significantly more abundant in yellow-fleshed loquats. In DWX-3x, WHZY, and HYWH No.1, β-cryptoxanthin laurate was the most abundant carotenoid, accounting for 28.57 %, 21.87 %, and 19.95 %, respectively. However, in HJWH No.1, β-carotene was the predominant carotenoid accounting for 38.93 % (Supplementary Fig. S7). The levels of β-carotene, violaxanthin-myristate-caprate, β-cryptoxanthin myristate, and lutein dilaurate were significantly higher in yellow-fleshed fruit pulp than that in white-fleshed. In addition, the overall carotenoid content was significantly higher in yellow-fleshed loquats (Supplementary Fig. S8). We further plotted a metabolic pathway diagram of the carotenoid contents (Supplementary Fig. S9), revealing the differences and changes in carotenoid contents between yellow-fleshed and white-fleshed triploid loquat.

Furthermore, we carried out a correlation analysis between color parameters and the identified carotenoids content within the four cultivars (Supplementary Table S9–S10). The Pearson correlation coefficients showed that the levels of β-carotene, violaxanthin-myristate-caprate, and lutein dilaurate had significant correlation with color parameters (Fig. 6D). The contents of these carotenoids were ranged from 0.94 μg/g·FW to 65.06 μg/g·FW, 4.24 μg/g·FW to 34.72 μg/g·FW, and 2.34 μg/g·FW to 10.65 μg/g·FW, accounting for 7.75 %-38.93 %, 11.06 %–22.21 %, and 3.21 %-14.28 % of the total carotenoids, respectively. Consequently, we infer that these three carotenoids contribute to the color differences between yellow- and white-fleshed triploid loquats (Supplementary Fig. S8 and Supplementary Table S8–S9). Subsequently, we analyzed the carotenoids-related pathways and found that differences in carotenoids content might be due to the conversion of lycopene to β-carotene (Supplementary Fig. S9).

### 3.8 Comparative analysis of lipidome in four triploid loquat cultivars

As detailed in Section 3.3, according to the enrichment of DAMs within the lipid class at the CT and FR stages, a higher concentration of lipids was found in white-fleshed triploid loquats. We further examined the lipidome of two yellow-fleshed triploid cultivars (DWX-3x and HJWH No.1) and two white-fleshed cultivars (WHZY and HYWH No.1) at the FR stage, utilizing a sample size of 36. The PCA analysis of lipidome (Fig. 6E) showed two distinct groups between the yellow- and white-fleshed triploid loquats. The OPLS-DA model (Supplementary Fig. S10) showed a Q^2^ > 0.921 and *P* < 0.05 for all comparison groups, suggesting the high reliability of the data for further analysis. A total of 27 lipid subclasses, comprising 570 individual lipids, were identified using UPLC-QTRAP-ESI-MS/MS methodology (Fig. 6F–H, Supplementary Table S11). The triglyceride (TG) class contained 193 lipids and was the subclass with the highest number of detected lipids (Supplementary Fig. S11). In the contents comparison of lipid subclasses, phosphatidylcholine (PC), phosphatidylethanolamine (PE), free fat acid (FFA), and phosphatidylserine (PS) were the most abundant lipid subclasses. Meanwhile, the PC, PE, and PS subclass belong to the class I of glycerol phospholipids (GP). The phosphatidylglycerol (PG), phosphatidylinositol (PI), PC, and PE accounted for the main proportion in triploid loquats and were significantly higher in the white-fleshed triploid loquats (Fig. 6G and Supplementary Fig. S12A and Table S12).

To identify key differentially accumulated lipids (DALs), comparative lipidomic analysis was performed between yellow- and white-fleshed loquat using the conditions of *P* value ≤ 0.05, VIP value ≥ 1, and fold change value ≥ 2 or ≤ 0.5. We identified 126, 89, 123, and 70 DALs in the comparison groups DWX-3x-FR *vs* WHZY-FR, DWX-3x-FR *vs* HYWH No.1-FR, HJWH No.1-FR *vs* WHZY-FR, and HJWH No.1-FR *vs* HYWH No.1-FR, respectively (Supplementary table S13 and Supplementary Fig S13). These DALs were mainly enriched in the class of GP and glycerolipids (GL) (Supplementary Fig S14). Among these DALs, five lipids significantly accumulated in four comparison groups, of which PI(18:3_16:0) (PubChem CID: 52927793) and PE(18:3_18:3) (PubChem CID: 9546735) were significantly higher in white-fleshed triploid loquats (Supplementary Fig S15 and Fig. S16), suggesting the two lipids PI(18:3_16:0) and PE(18:3_18:3) could be as lipid markers for yellow and white-fleshed triploid loquats (Supplementary Fig. S16B).

## 4 Discussion

### 4.1 Differences in sugar content and flavor of organic acids between yellow- and white-fleshed triploid loquats

The range of different metabolites provides a specific olfactory stimulus that offers unique properties. Sucrose, glucose, and fructose are the most predominant sugar components in loquat fruit, but they have a different sweet taste, and fructose is sweeter than the other two. A previous study has established a sweetness index to calculate sweetness (Keutgen and Pawelzik, 2007). In this study, we determined by HPLC that white-fleshed triploid loquats had higher levels of fructose and lower levels of glucose and sucrose than those of yellow-fleshed triploid loquats, and the calculated sweetness index was not significantly different. Previously, Chen et al. (2010) found that the white-fleshed variety Ninghaibai has a higher fructose content than the yellow-fleshed loquat Dahongpao, with no significant differences in glucose and sucrose contents, which is consistent with our results. Therefore, the difference in flavor may not be significant, and the difference in taste is mainly due to different organic acids or other metabolites.

Analysis of organic acids showed that white-fleshed loquats had a significantly lower malic acid content than yellow-fleshed loquats. Consumers prefer the white-fleshed loquat cultivars because their fruits have a comparatively better flavor. Malic acid was the most dominant acid, accounting for 62.1 % of the total acid content. Furthermore, cluster analysis showed that white-fleshed varieties were classified into low-acid groups (Chen et al., 2008). Previous studies on apple fruit taste have indicated that fruit acidity plays an important role in the improvement of fruit taste, but high fruit acidity can lead to an unpleasant taste (Liao et al., 2021). Therefore, the fruits of white-fleshed triploid loquats taste better, which might be due to the high fructose and low malic acid content.

### 4.2 Differences in DAMs between yellow- and white-fleshed triploid loquats

We compiled flavor-related metabolites from previous studies, compared them with data obtained from our experimental metabolome, and identified 34 flavor-related metabolites, of which 10 were significantly different. First, we analyzed the flavor metabolites of the saccharide and alcohol classes. Melibiose and gluconic acid, which are the only significantly different metabolites in this class, are common industrially used food additive ingredients (Novotny et al., 2019). The melibiose content in yellow-fleshed triploid loquats was higher than that in white-fleshed triploid loquats. However, the content of gluconic acid in white-fleshed triploid loquats was higher.

Although most amino acids are tasteless, they provide various aromas to impart flavor to fruits (Xu et al., 2022). In our study, l-lysine and l-glutamine were significantly higher in yellow-fleshed triploid loquats than in white-fleshed loquats, which is different from that reported in diploid loquats (Zou et al., 2020). Phenolic acid, a phenolic compound, has sour, bitter, astringent, and phenol-like flavors. It is also an important secondary metabolite, which significantly affects the overall quality of the fruit, including taste, nutritional attributes, and appearance. In this work, we detected three organic acids, including 4-caffeoylquinic acid, cynarin, vanillin, and villoy caffeoyl tartaric acid in the flesh of triploid loquats and their contents were significantly higher in white-fleshed loquats than in yellow-fleshed loquats. Previously, these three organic acids have been identified in tomatoes and are related to fruit flavor (Bakir et al., 2020).

Tannins have a bitter taste and cholesterol-lowering effects (Tebib et al., 1994). In this study, secondary metabolite analysis showed that the tannin content was relatively high in white-fleshed triploid loquat. Meanwhile, we found that catechin was more abundant in triploid white loquat, whereas narirutin showed an opposite trend. Previously, catechins were important in providing a rich flavor and astringent taste to tea and have various health-promoting properties including relaxation and neuroprotection. Narirutin is indirectly associated with the flavor and quality of citrus fruits (Deterre et al., 2021).

### 4.3 Metabolic dynamics of flavor between yellow- and white-fleshed triploid loquats

To better understand the dynamics of flavor components, we analyzed the key DAMs between yellow-fleshed and white-fleshed triploid loquats in ripening fruit flesh. This enabled us to identify the key sugar components, organic acid metabolites, and their pathways (Fig. 5B). Among them, fructose, sucrose, and glucose levels gradually increased with fruit development and reached maximum values at the FR stage. Similar results have been reported in other fruit trees of the Rosaceae family, such as apples (Liao et al., 2021), apricots, plumcots, plums, and peaches (Bae et al., 2014). In our study, yellow-fleshed triploid loquat fruit contains more sucrose and glucose but relatively less fructose at the CT and FR stages. Similarly, a previous report has also demonstrated significant variations in different sugar components during the CT stage in apples (Zhu et al., 2013). Therefore, we speculate that the differences in sugar components were caused by the CT stages in yellow- and white-fleshed triploid loquats.

In our work, malic acid was the predominant organic acid in triploid loquat fruit and its content gradually decreased with fruit maturity, similar to that of a previous study (Chen et al., 2008). PEP, which is upstream of the metabolic pathway of malate, showed a changing trend similar to that of malic acid, suggesting the possibility of significant changes in the upstream pathway to PEP. Similarly, a previous study has also shown a decreasing trend in the organic acid metabolic pathway in apples (Fernie et al., 2004).

### 4.4 Screening of other key metabolites

To better understand the differences between other metabolites in triploid loquats, we identified other key metabolites, which might provide metabolic markers for breeding improved varieties. As shown in Fig. 5A-B, four metabolites, including trigonelline, (S)-2-(4-Aminobutanamido)-3-(1-methyl-1H-imidazol-5-yl), PE(18:3/18:3 + O3), and cimicifugic acid L, were screened unique to WHZY during the FR period, while trigonelline was not detected in HJWH No.1. Trigonelline has been reported to have hypoglycemic, hypolipidemic, neuroprotective, and antimigraine properties (Zhou et al., 2012) and was only detected in WHZY. Furthermore, (S)-2-(4-Aminobutanamido)-3-(1-methyl-1H-imidazol-5-yl) propanoic acid, also known as anserine, can improve blood flow in the brain and memory functions in elderly adults (Ding et al., 2018). PE(18:3/18:3 + O3) is a phosphatidylethanolamine, the receptor of protein kinase C, and is essential to induce cell activation and proliferation, differentiation, motility, and survival (Isakov, 2018). Cimicifugic acid L has beneficial vasoactive effects (Noguchi et al., 1998).

### 4.5 Variations of carotenoids and lipids in different colored triploid loquats

Carotenoids, which are responsible for a variety of colors ranging from yellow to red, are widely participated in fruit color and quality (Cai et al., 2019). In our work, the total carotenoids content in yellow-fleshed triploid loquat fruit flesh is significantly higher than in white-fleshed triploid loquat. In the flesh of triploid cultivars, we identified six key carotenoids, including β-cryptoxanthin laurate, β-carotene, violaxanthin-myristate-caprate, β-cryptoxanthin oleate, β-cryptoxanthin myristate, and lutein dilaurate. Similarly, previous studies have indicated that carotenoid concentrations in yellow-fleshed diploid loquats were significantly higher than those in white-fleshed diploid loquats (Cai et al., 2019, Zhou et al., 2007). A previous report has shown that β-cryptoxanthin and β-carotene are the most abundant carotenoids in diploid loquat flesh (Zhou et al., 2007). Meanwhile, *trans*-β-cryptoxanthin, *trans*-β-carotene, and 5,8-epoxy-β-carotene were identified in diploid fruit flesh (Hadjipieri et al., 2017).

Lipids play crucial roles in fruit development, seed maturation, and stress response in plants. For example, the most abundant lipid classes in nuts and oily fruit are PC, PE, PI, phosphatidic acid (PA), and phosphatidylglycerol (PG) (Jardim et al., 2023). In our study, we identified 27 lipid subclasses, including 570 lipids in triploid loquat fruit flesh, of which the most abundant lipid classes were PC, PE, FFA, and PS. These results provide new insights into lipid composition in triploid loquats. Therefore, we investigate the lipid composition that might play an important role in the fruit development of the triploid loquats.

## 5 Conclusions

In this study, we used widely targeted metabolomic analyses and HPLC to investigate the metabolite diversity in yellow- and white-fleshed triploid loquats during fruit development. We identified key DAMs to elucidate the variations in flavor and substance composition. Furthermore, we performed carotenoids and lipidomic analyses to expand metabolomic data. Compared with yellow-fleshed triploid loquat, the carotenoids contents are significantly downregulated, but the contents of most glycerolphospholipids are increased in the fruits of white-fleshed varieties. Our study provides further insights into the underlying mechanisms of fruit quality differences in triploid loquats. These results also provide metabolic markers for breeding improved varieties of triploid loquats and enhance our understanding of fruit flavor and the biosynthesis of secondary metabolites in loquats.

## CRediT authorship contribution statement

**Xinya Liu:** Conceptualization, Methodology, Data curation, Supervision, Writing – original draft. **Liqin Song:** Data curation, Methodology, Writing – original draft. **Baogui Xue:** Methodology, Software. **Zhuoheng Chi:** Investigation. **Yuan Wang:** Investigation. **Songqin Wen:** Investigation. **Wenjuan Lv:** Data curation. **Qiankun Hu:** Data curation. **Qigao Guo:** Funding acquisition. **Shuming Wang:** Writing – review & editing. **Di Wu:** Writing – review & editing. **Guolu Liang:** Funding acquisition, Validation. **Danlong Jing:** Conceptualization, Funding acquisition, Validation, Writing – review & editing.

## Declaration of competing interest

The authors declare that they have no known competing financial interests or personal relationships that could have appeared to influence the work reported in this paper.
