# Supplementary material for: Organic acid and sugar components accumulation and flavor associated metabolites dynamic changes in yellow- and white-fleshed seedless loquats (Eriobotrya japonica)
Source: Food Chem X. 2023 Dec 6;21:101046. doi: 10.1016/j.fochx.2023.101046 (PMC10762357; doi:10.1016/j.fochx.2023.101046)
Supplement: Supplementary data 1 [file mmc1.docx]

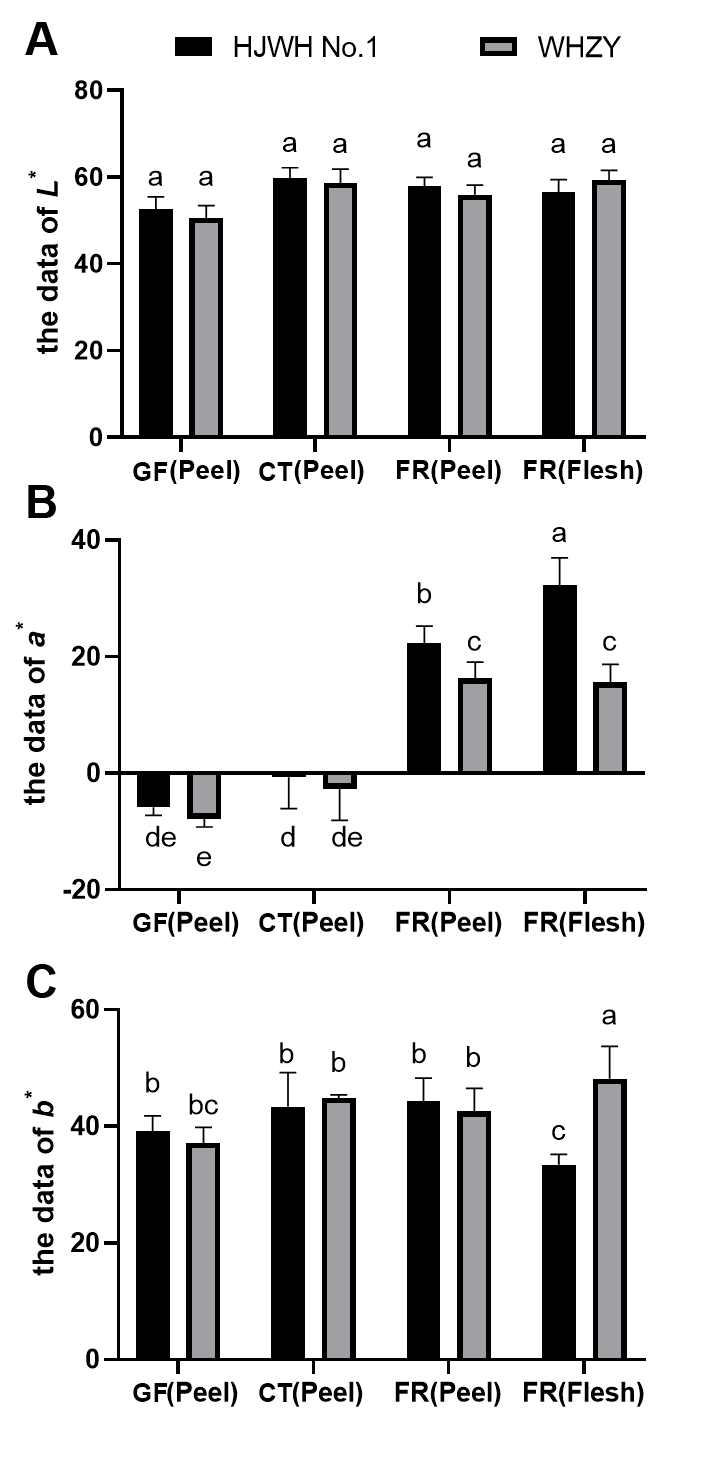


# Supplementary Fig. S1 The *L*^*^ (A), *a^*^*(B), *b^*^*(C) values of peel and flesh at different periods of loquat development. Each data represents the mean of 3 measured values. The bars represent standard deviation (SD) (n=3). Different letters indicate statistically significant differences using one-way ANOVA followed by Duncan's multiple range test.

Note: *L*^*^ indicates brighter and darker colors; higher and lower values indicate whiter and darker colors, respectively. *a*^*^ indicates red and green; higher and lower values indicate redder and greener colors, respectively. *b*^*^ indicates yellow and blue; higher and lower values indicate yellow and blue colors, respectively.


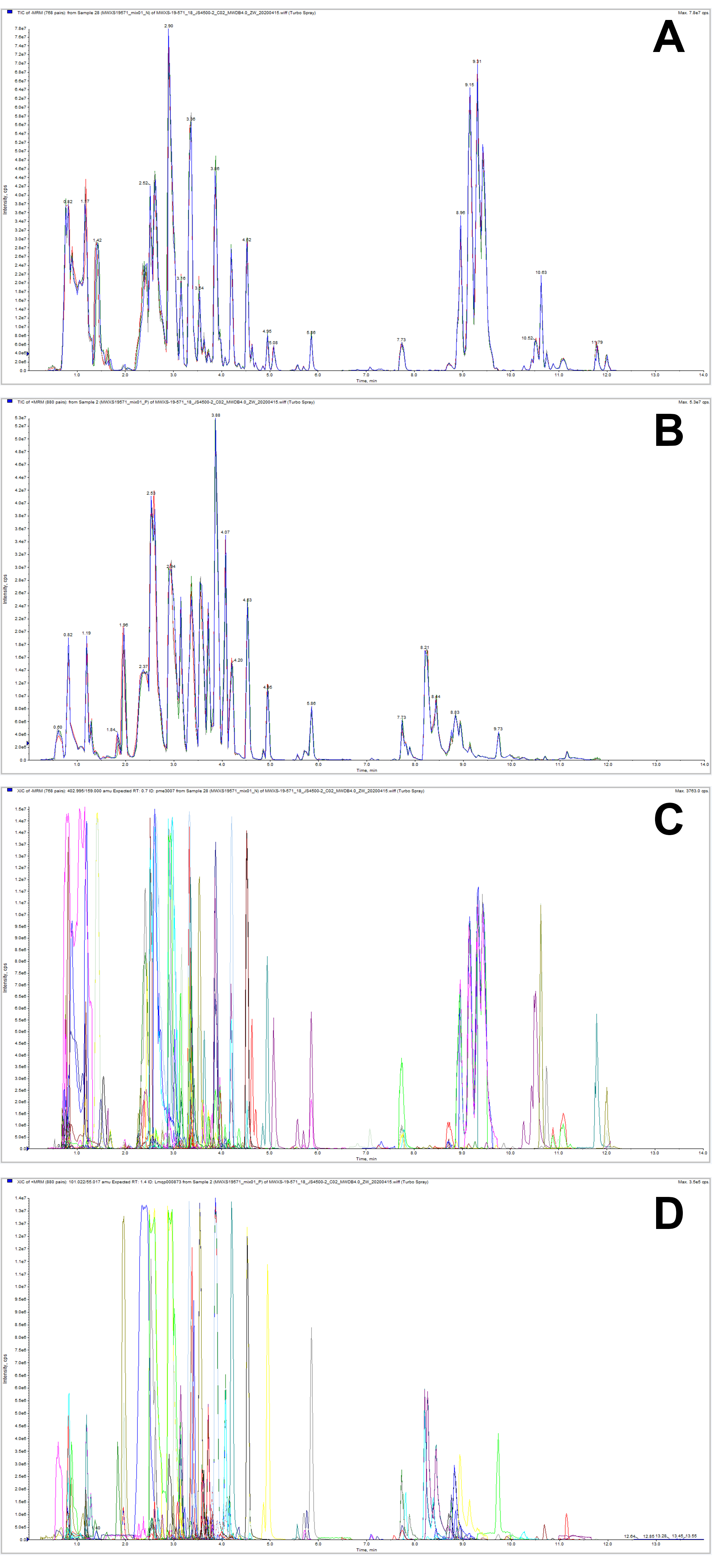


# Supplementary Fig. S2 (A–B) Total ions current (TIC) overlapping map of QC samples mass spectrometry results. The abscissa is the retention time of the metabolite detection. The ordinate is the ion current intensity of the ion detection (the intensity units are counts per second (cps). (C–D) A multi-peak detection plot of the metabolites in the multiple reaction monitoring mode. (A, C) in negative ion mode, (B, D) in positive ion mode.


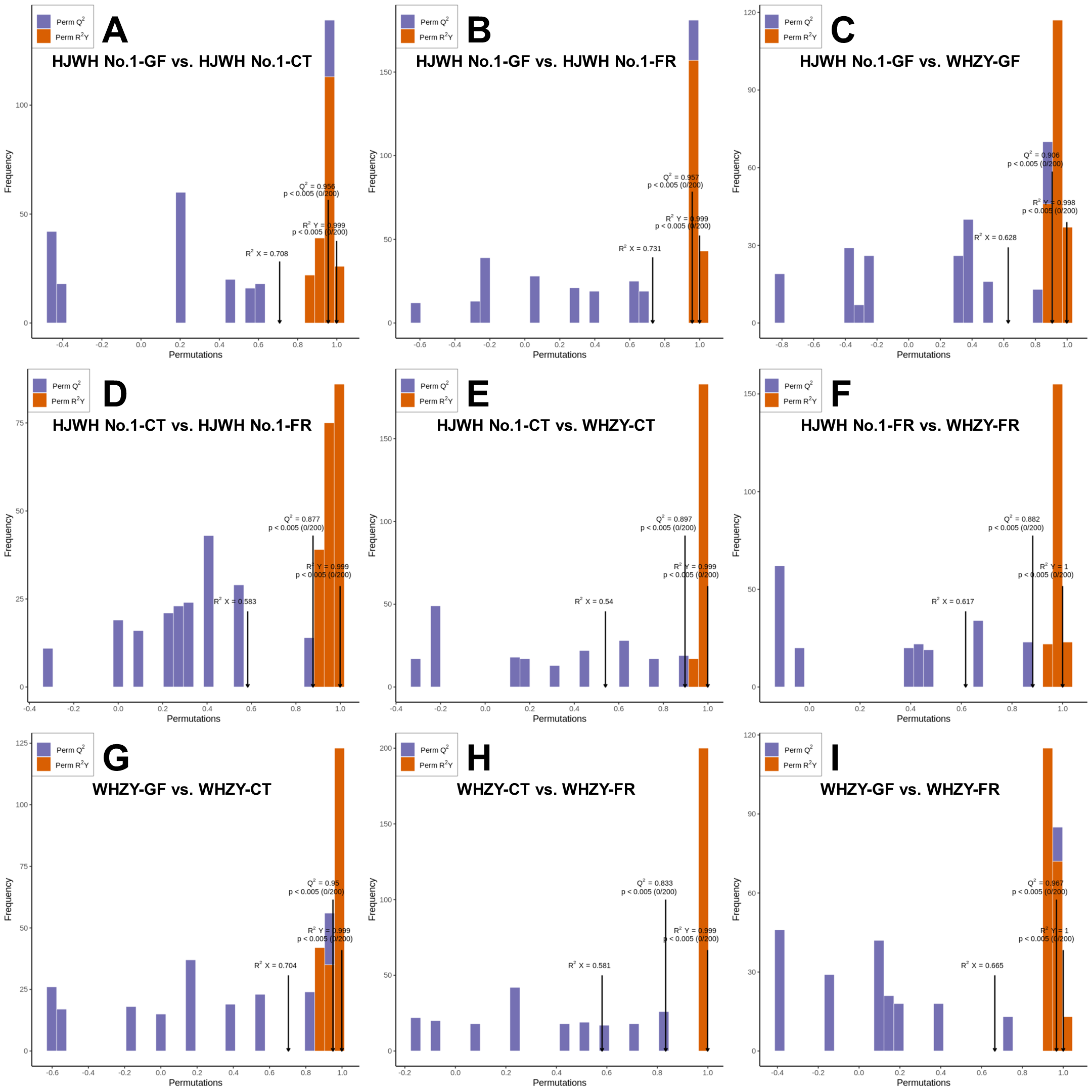


# Supplementary Fig. S3 OPLS-DA model arrangement permutations diagram.

Note: The horizontal coordinate indicates the accuracy of the model, and the vertical coordinate is the frequency of the model classification effect, that is, this model performs 200 random permutation experiments on the data; if *p*=0.02 for Q^2^, it means that a total of 4 random grouping models have better prediction ability than this OPLS-DA model in this permutation test, and if *p* = 0.545 for R^2^Y, it means that 109 random grouping models have a better interpretation of the Y matrix than this OPLS-DA model in this permutation test. If *p* = 0.545 for R^2^Y, it means that there are 109 random grouping models in the permutation test whose explanation rate of the Y matrix is better than this OPLS-DA model. The model is best at *p*<0.05.


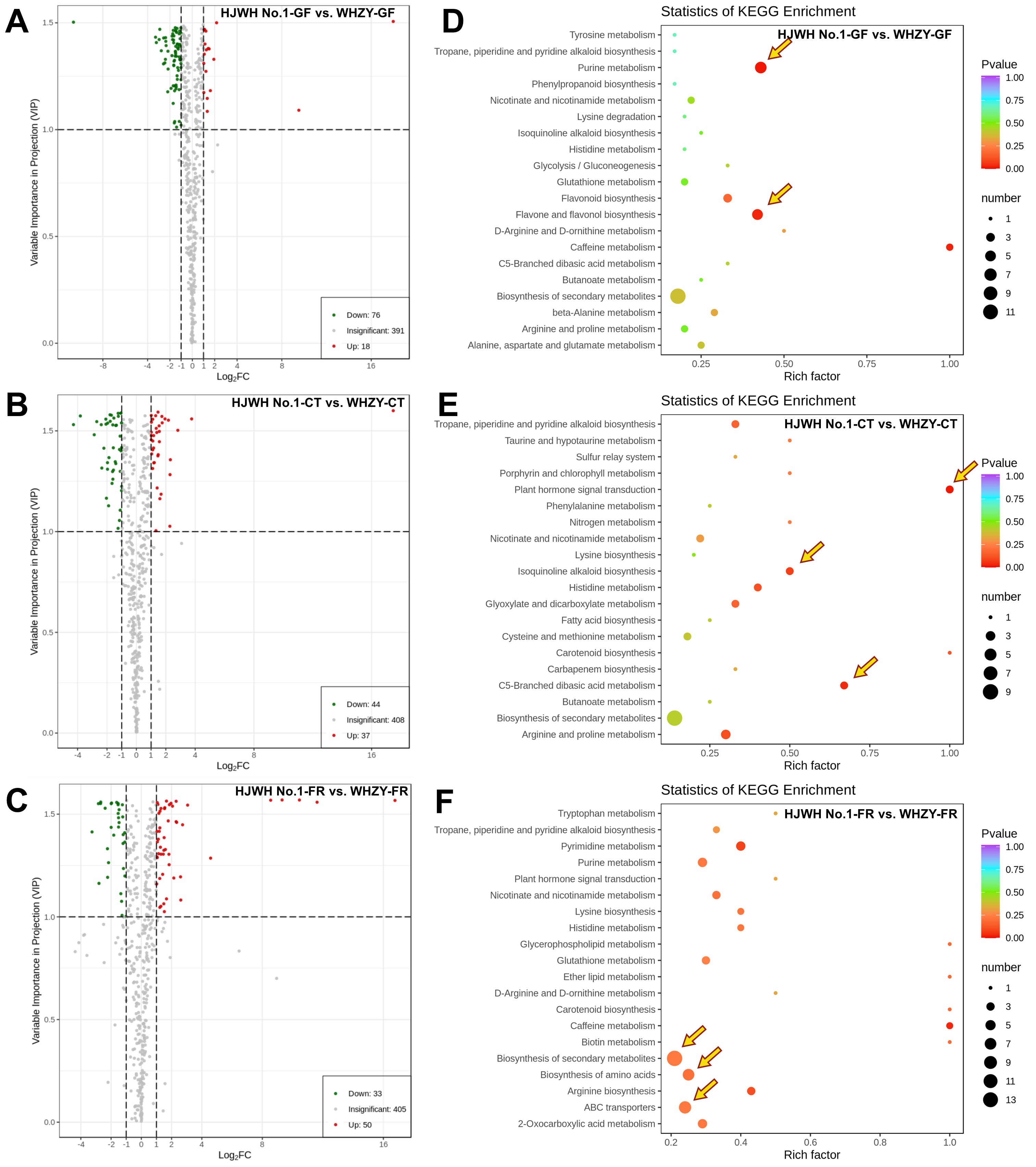


# Supplementary Fig. S4 DAMs between HJWH No.1 and WHZY. (A, B, C) Volcano plot of the 489 metabolites identified. DAMs were defined as metabolites with a fold change ≥ 1.0 or ≤ 0.5 in HJWH No.1 compared to WHZY. A threshold of VIP≥1.0 was used to separate DAMs from unchanged metabolites. The red data points represent the up-regulation of the metabolite in the comparison, whereas the opposite green represents the down-regulation. (D, E F) KEGG classification of DAMs between HJWH No.1 and WHZY. (A, D), (B, E), (C, F) are for HJWH No.1 and WHZY`s DAMs in the GF, CT, and FR stages, respectively.


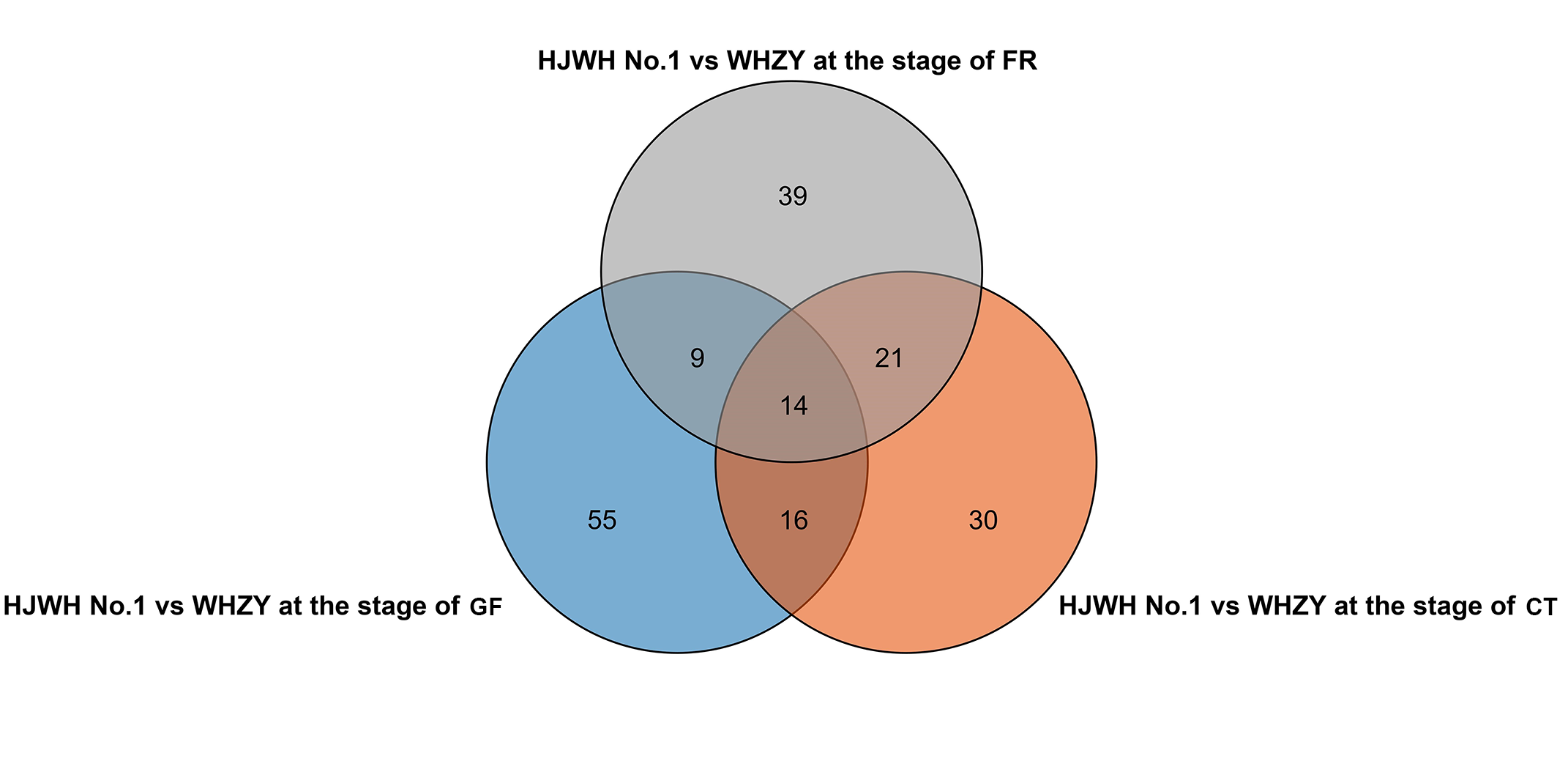


# Supplementary Fig. S5 Venn diagram of the number of DAMs between three groups.


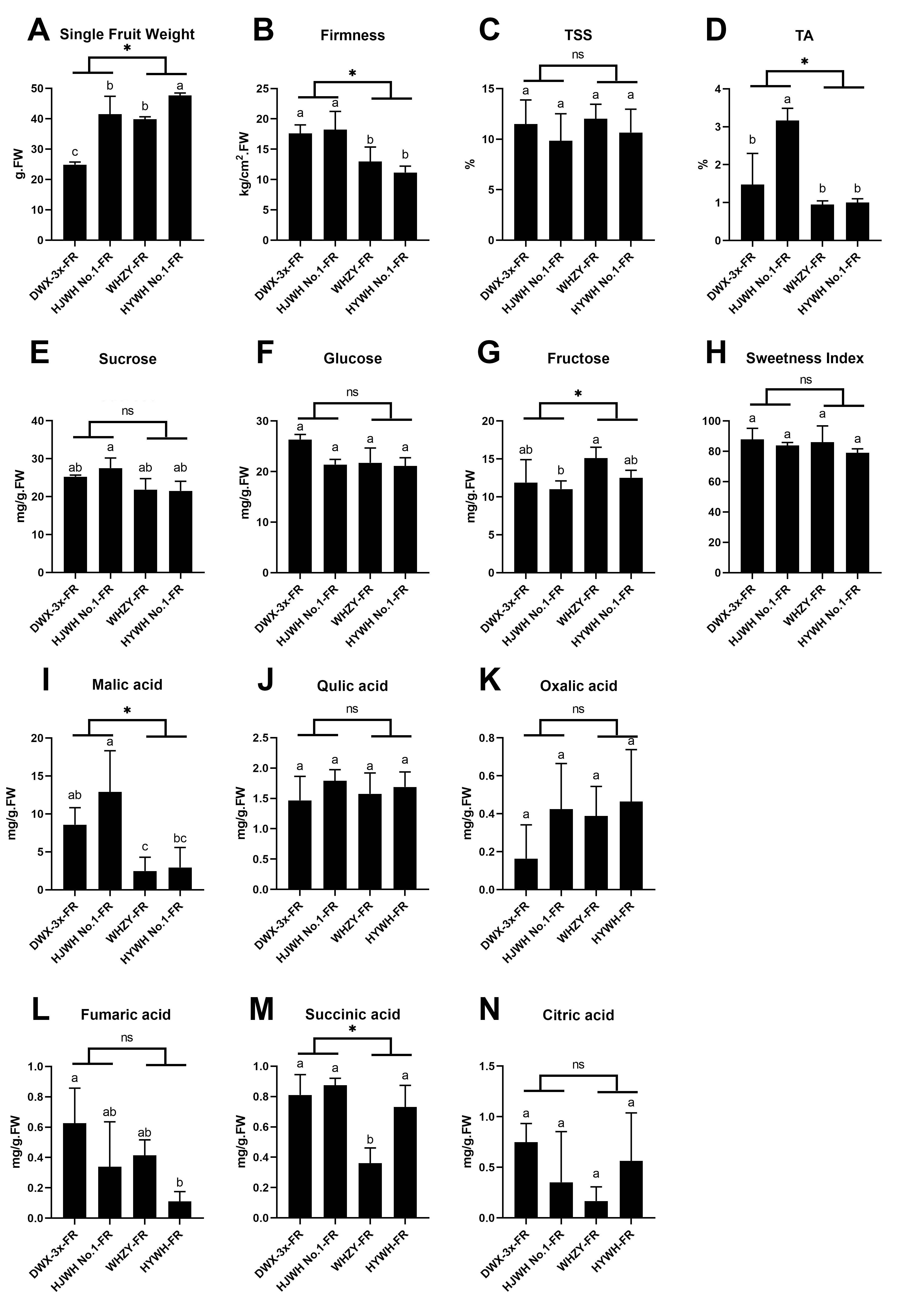


# Supplementary Fig. S6 (A-N) The histograms of the parameters related to fruit quality of two yellow-fleshed seedless triploid loquat cultivars DWX-3x and HJWH No.1 and two white-fleshed seedless triploid loquat cultivars WHZY and HYWH No.1 at their FR stage. (A) and (B) represent the single fruit weight and firmness of the single fruit of the 4 cultivars, respectively. (C) TSS represents the total soluble solids. (D) TA represents titratable acid. (E-G, I-N) The bar charts of 3 sugar components and 6 organic acids detected by HPLC at the FR stages of 4 cultivars. (H) The Sweetness Index of DWX-3x, HJWH No.1, WHZY and HYWH No.1 at FR stage. The data contained 3 replicates. Data are presented as mean ± standard deviation (SD). Data within different groups of single fruit cultivar groups were analyzed by one-way ANOVA followed by Duncan's multiple range test to test for significant differences between means (*P* < 0.05). Different letters above the bars indicate significant differences (*P* < 0.05). Data within different groups of different color were analyzed by Independent Samples t-test. The symbol * represents the selected groups differed significantly after a two-way analysis of variance (*P* < 0.05), and ‘ns’ represents there is no significant.


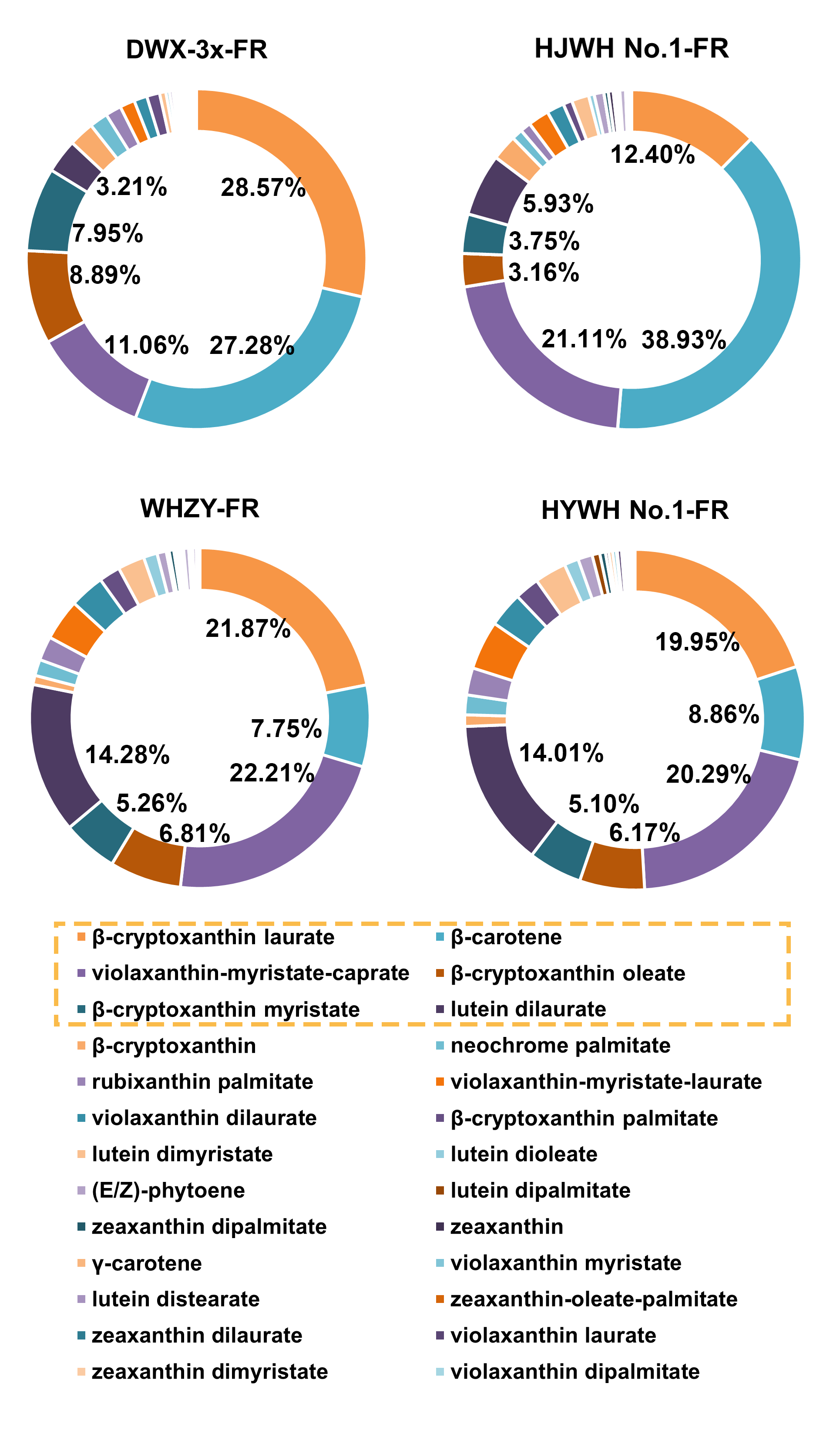


# Supplementary Fig. S7 The Fan diagram of the distribution ratio of the different carotenoids content of the four cultivars at the FR stages.


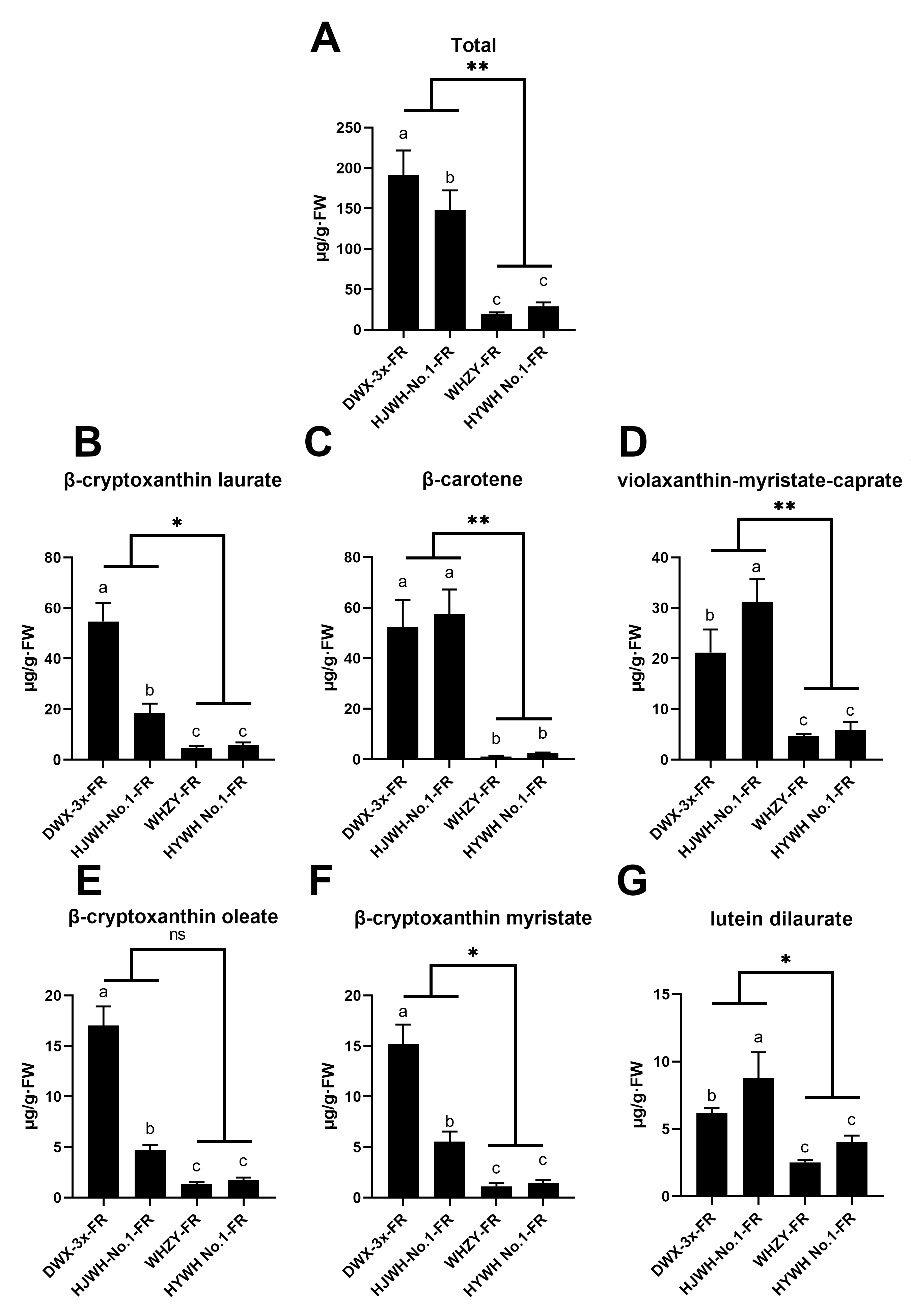


# Supplementary Fig. S8 (A) The histogram of the total carotenoids content in 4 seedless loquat cultivars at the FR stage. (B-G) The histogram of the content of different single carotenoid in 4 seedless loquat cultivars at the FR stage. The data contained 3 replicates. Data within different groups of single fruit cultivar groups were analyzed by one-way ANOVA followed by Duncan's multiple range test to test for significant differences between means (*P* < 0.05). Different letters above the bars indicate significant differences (*P* < 0.05). Data within different groups of different color were analyzed by Independent Samples t-test. The symbol * and ** represents the selected groups differed significantly (*p* < 0.05) and extremely significantly (*p* < 0.01) respectively after a two-way analysis of variance, and ‘ns’ represents there is no significant.


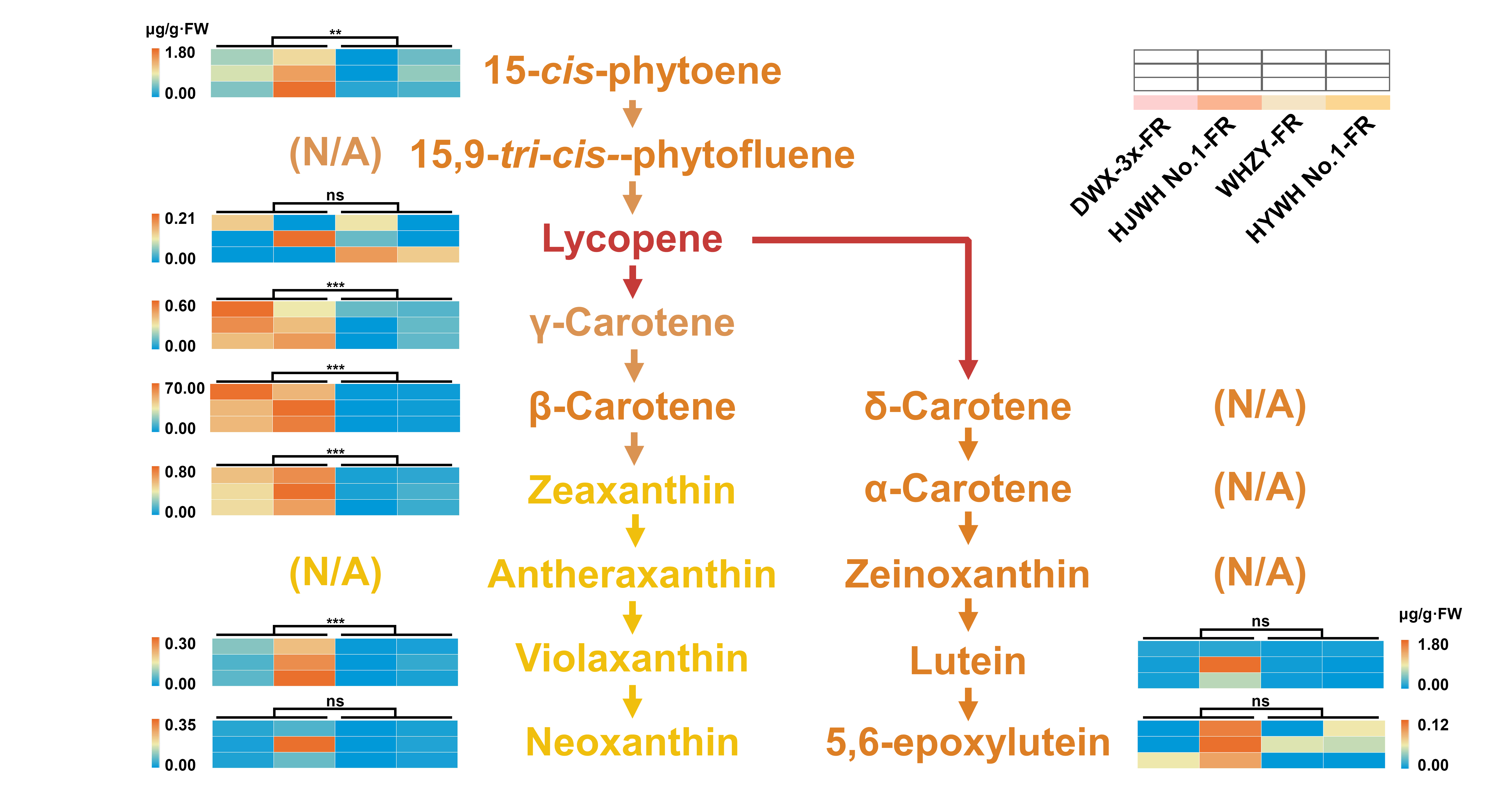


# Supplementary Fig. S9 The carotenoid metabolism roadmap. The depth of the color blocks in the heat map represents the content of carotenoids. Data within different groups of different color were analyzed by Independent Samples t-test. The symbol ‘(N/A)’ indicates ‘Not Acceptable’, that the substance has not been detected or the sample does not contain.


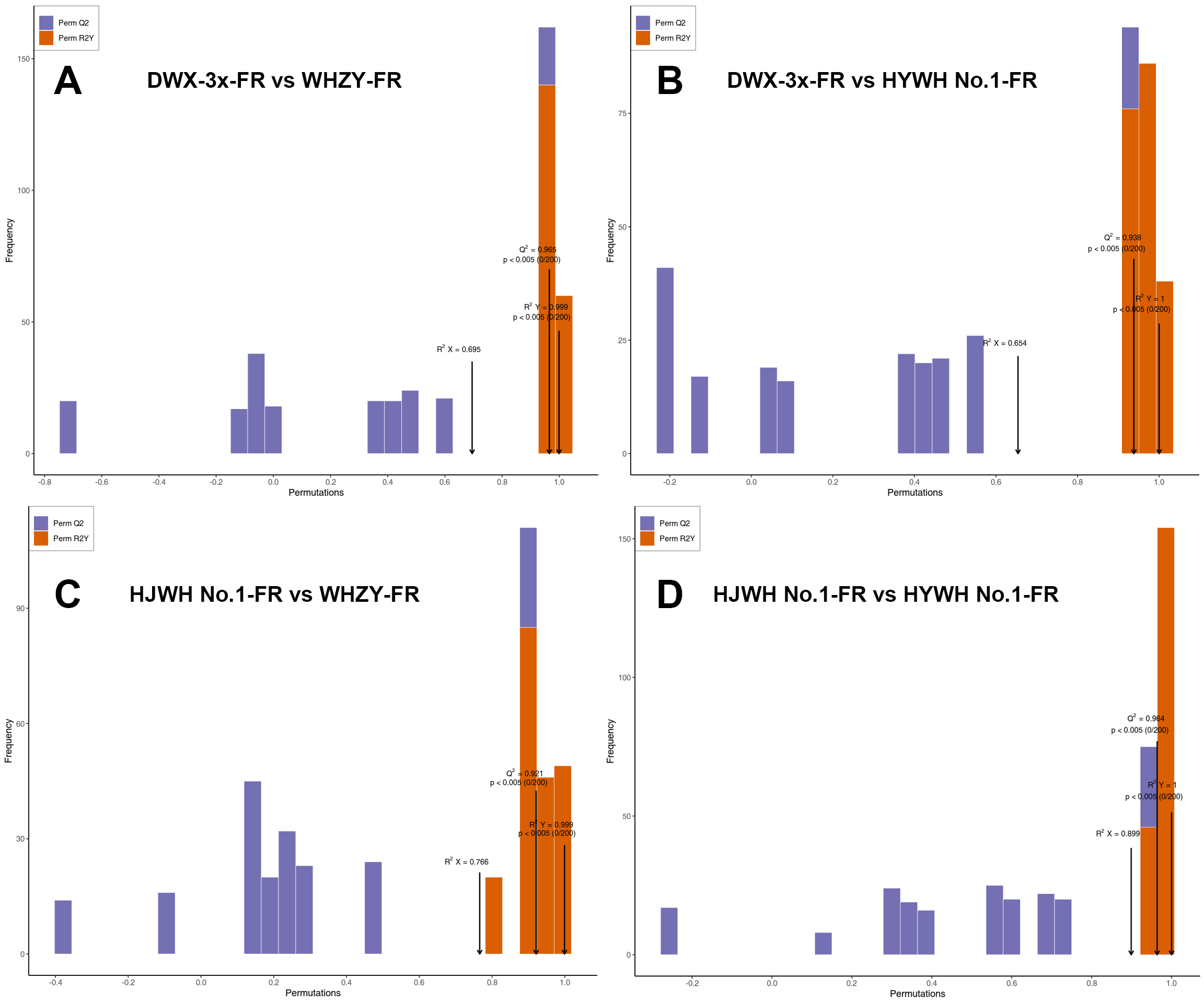


# Supplementary Fig. S10 (A-D) OPLS-DA model arrangement permutations diagram of the lipidomic data.


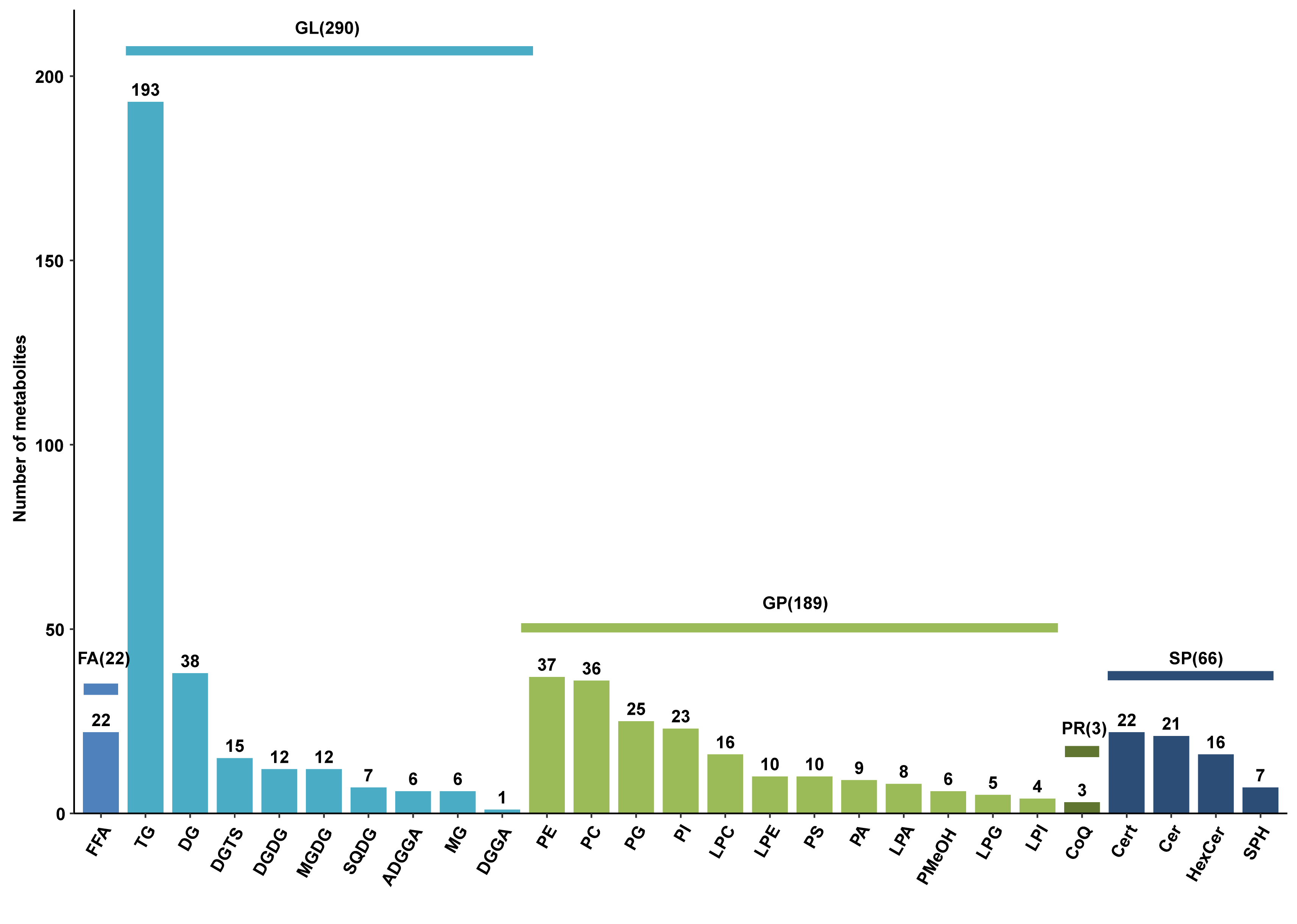


# Supplementary Fig. S11 Histogram of the lipid subclasses and identification of the number of lipids in each subclass. The Full names of lipid class I and class II are shown in the Supplementary Table S11.


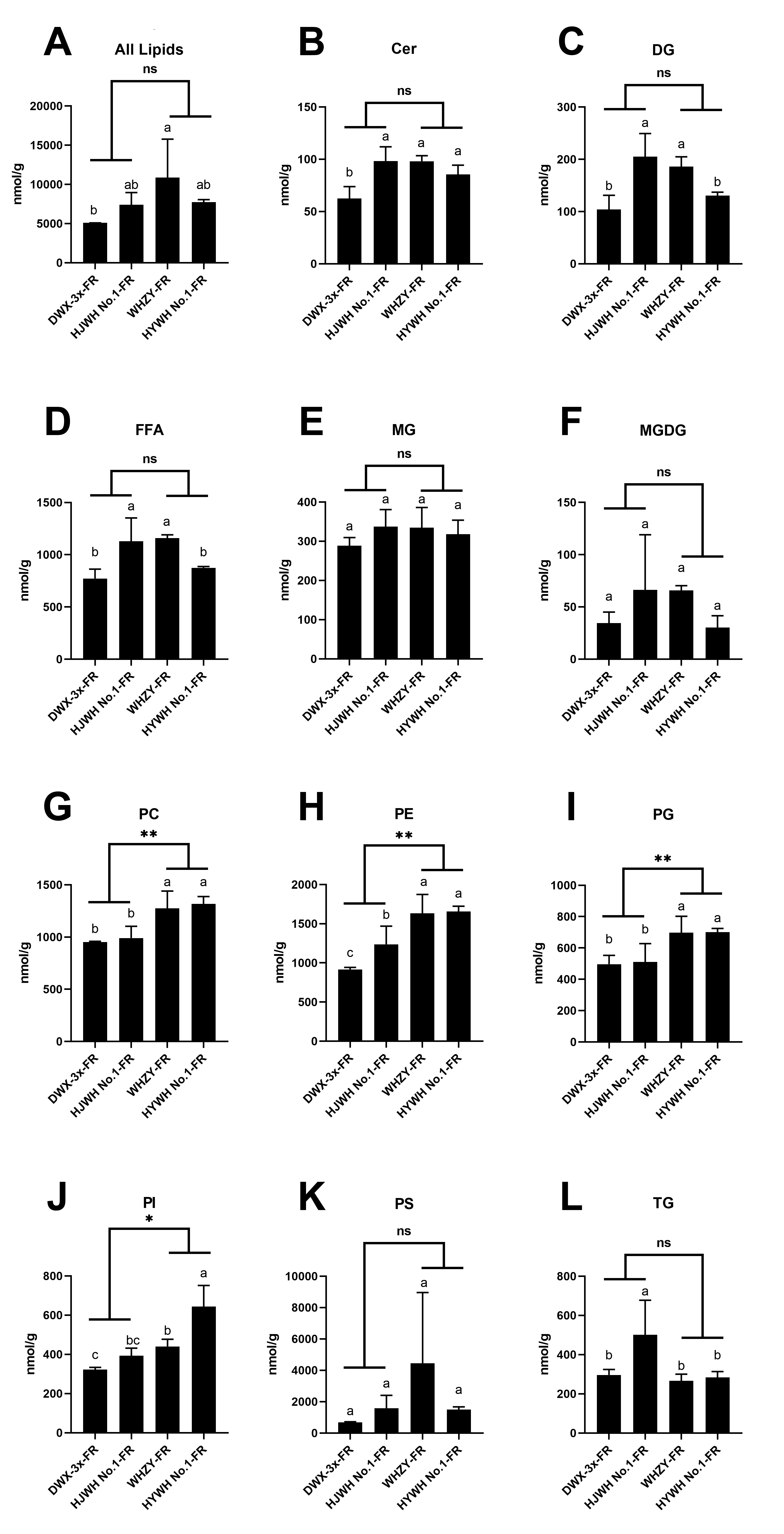


# Supplementary Fig. S12 (A) Total lipid content of four cultivars during the FR period. (B-L) The content of different lipid subclasses during the FR period in four cultivars. Data within different groups of single fruit cultivar groups were analyzed by one-way ANOVA followed by Duncan's multiple range test to test for significant differences between means (*P* < 0.05). Different letters above the bars indicate significant differences (*P* < 0.05). Data within different groups of different color were analyzed by Independent Samples t-test. The symbol * represents the selected groups differed significantly after a two-way analysis of variance (*P* < 0.05), and ‘ns’ represents there is no significant. The symbol * and ** represents the selected groups that differed significantly (*P* < 0.05) and extremely significantly (*P* < 0.01) respectively after a two-way analysis of variance, and ‘ns’ represents there is no significant.


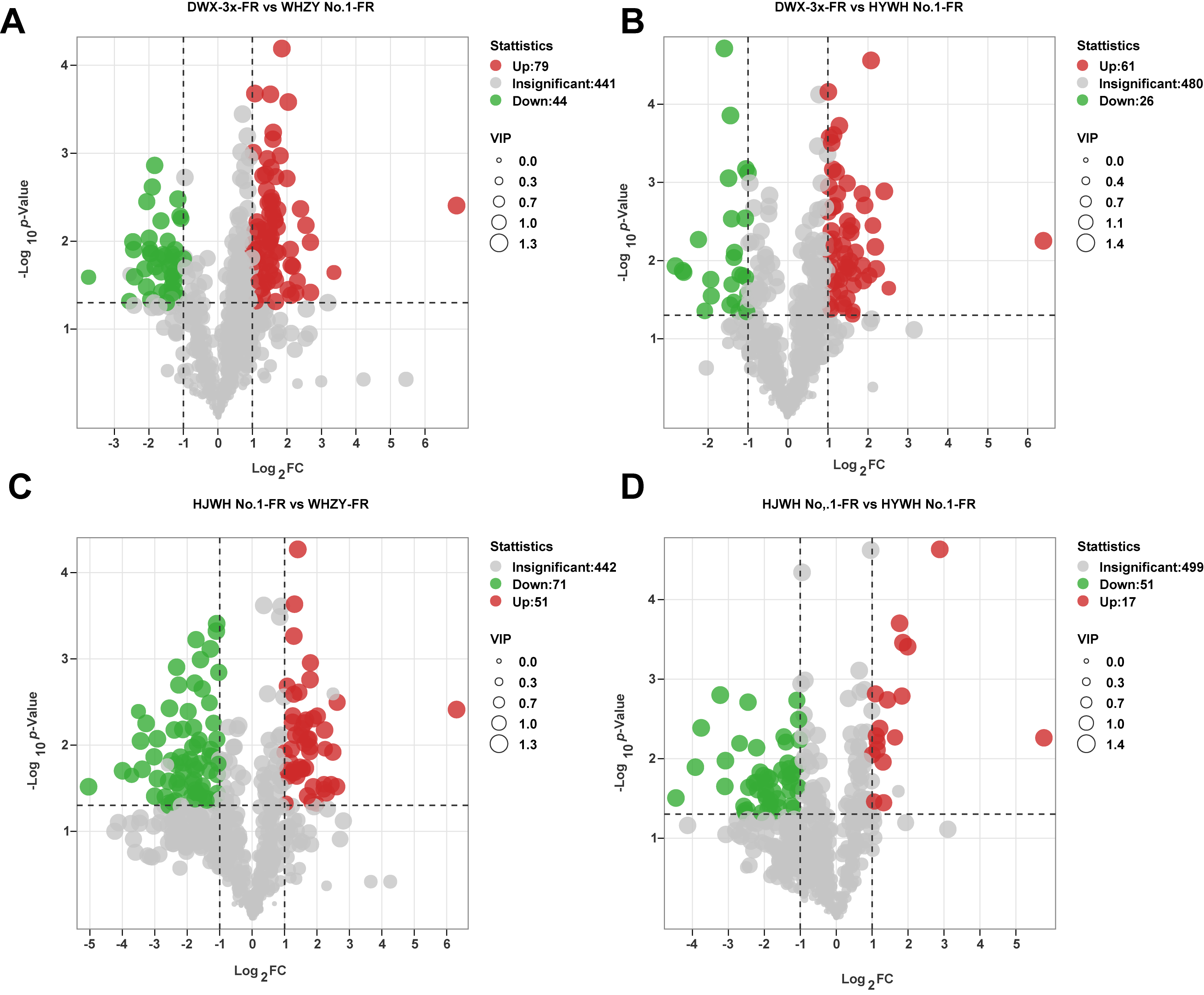


# Supplementary Fig. S13 （A-D）Differential lipid volcano maps after screening by condition. The green color in the chart indicates downward regulation and the red color indicates upward regulation. The size of the circle represents the size of the VIP value.


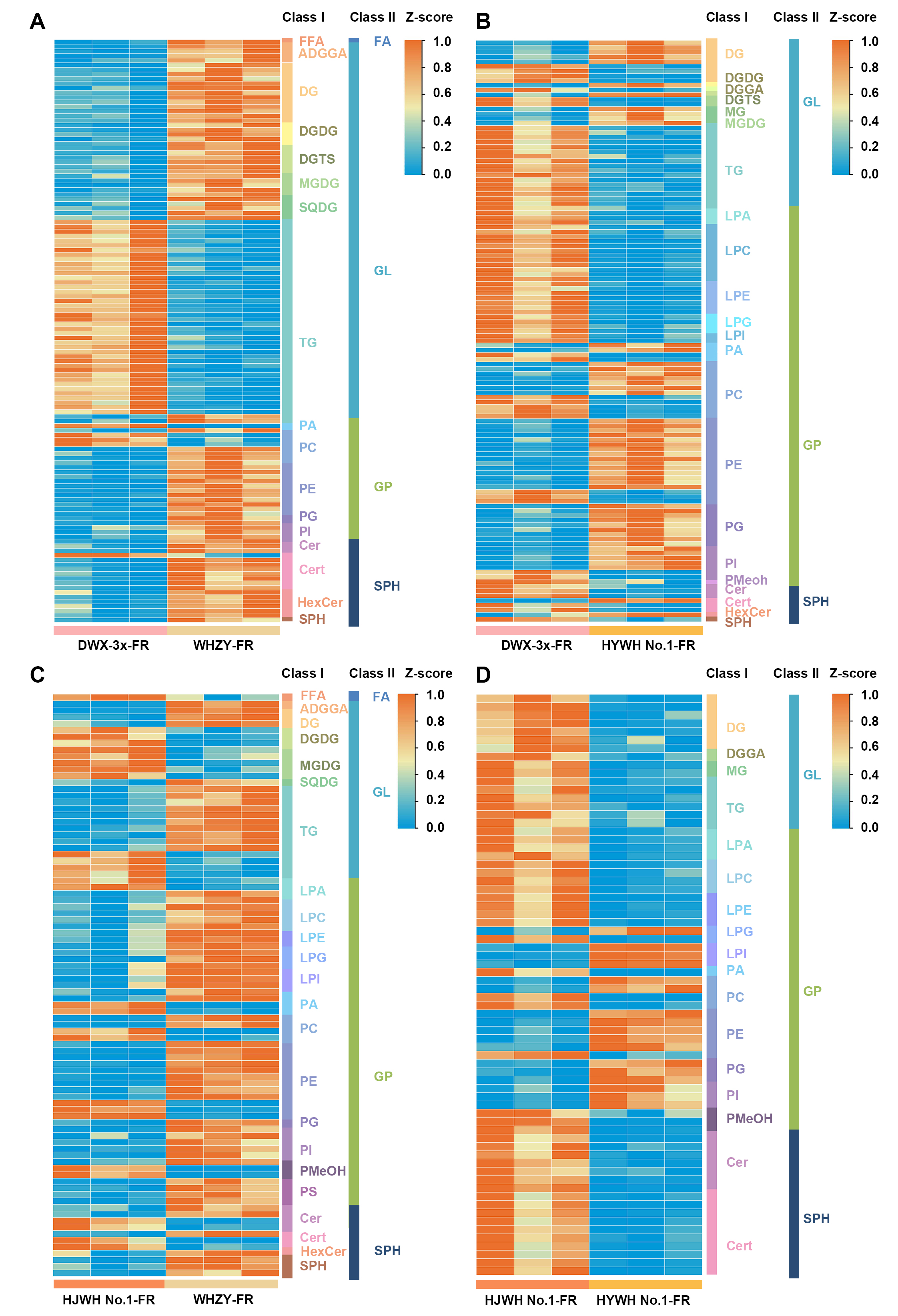


# Supplementary Fig. S14 Different comparison group of lipidome. The data is normalized by min-max method.


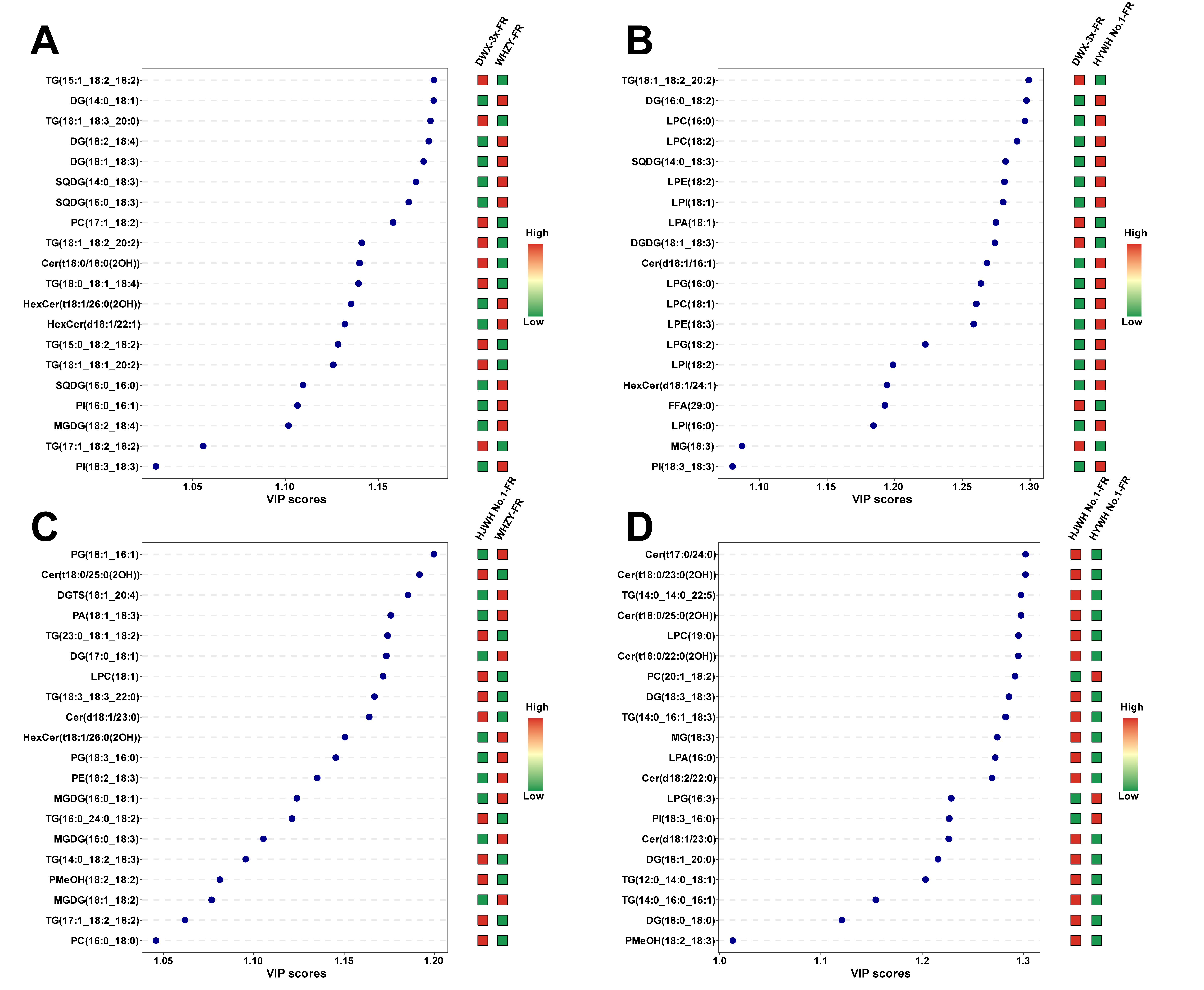


# Supplementary Fig. S15 The VIP score map and heatmap of differential accumulation lipids between different comparison group.


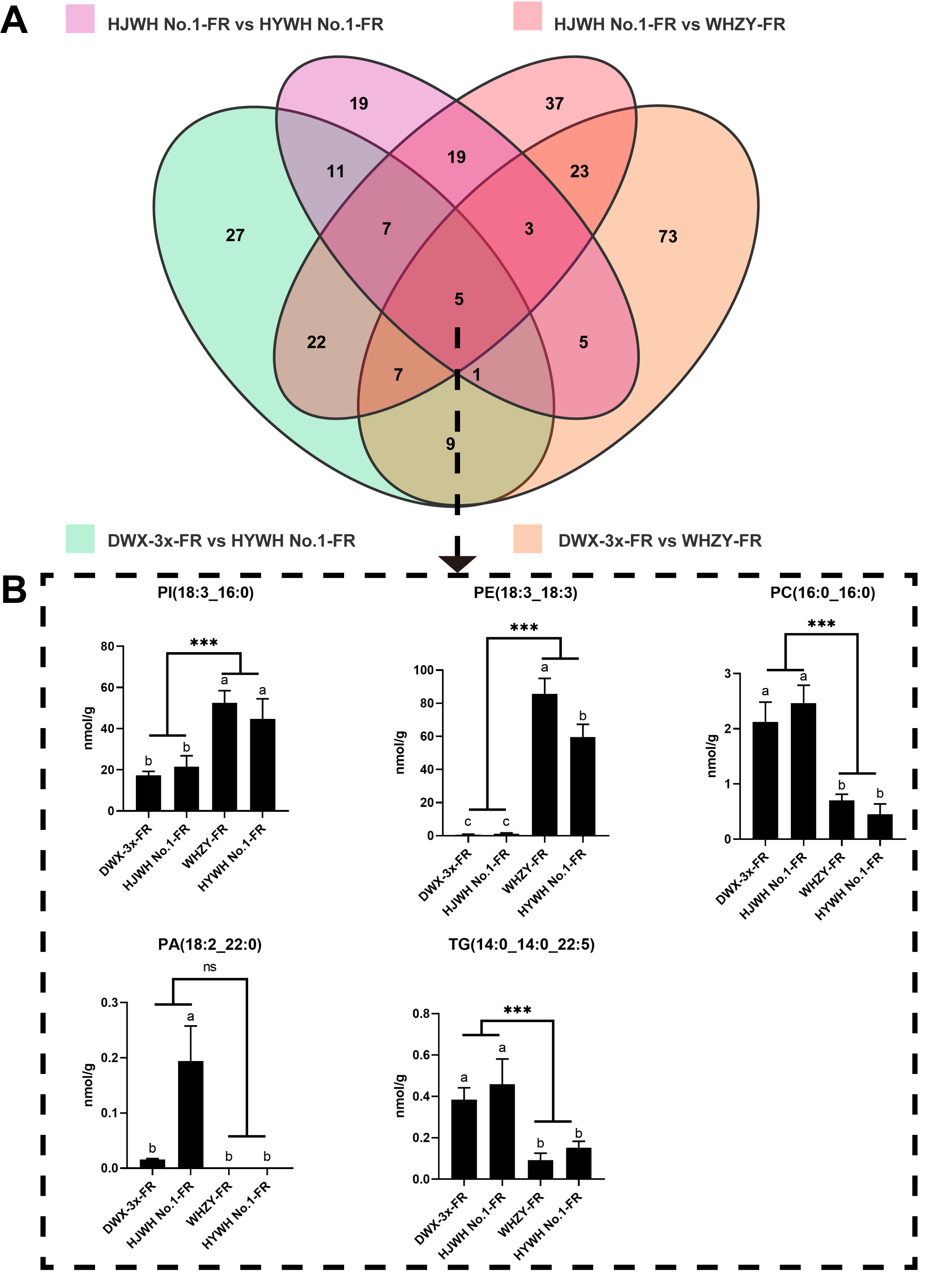


# Supplementary Fig. S16 (A)The Venn chart of different comparison groups of lipidomic. (B) The column chart of screened differential accumulation lipids content. Data within different groups of single fruit cultivar groups were analyzed by one-way ANOVA followed by Duncan's multiple range test to test for significant differences between means (*P* < 0.05). Different letters above the bars indicate significant differences (*P* < 0.05). Data within different groups of different color were analyzed by Independent Samples t-test. The symbol *, **and *** represents the selected groups differed significantly (*P* < 0.05), extremely significantly (*P* < 0.01) and *P* < 0.001, respectively after a two-way analysis of variance, and ‘ns’ represents there is no significant and ‘ns’ represents there is no significant.
